# Small nucleolar RNA host gene 18 controls vascular smooth muscle cell contractile phenotype and neointimal hyperplasia

**DOI:** 10.1093/cvr/cvae055

**Published:** 2024-03-18

**Authors:** Kaiyuan Niu, Chengxin Zhang, Mei Yang, Eithne Margaret Maguire, Zhenning Shi, Shasha Sun, Jianping Wu, Chenxin Liu, Weiwei An, Xinxin Wang, Shan Gao, Shenglin Ge, Qingzhong Xiao

**Affiliations:** William Harvey Research Institute, Faculty of Medicine and Dentistry, Queen Mary University of London, John Vane Science Centre, Charterhouse Square, London, EC1M 6BQ, UK; Department of Otorhinolaryngology, Third Affiliated Hospital of Anhui Medical University, No. 390, Huaihe Road, LuYang District, Hefei, Anhui, 230061, PR China; Department of Cardiovascular Surgery, First Affiliated Hospital of Anhui Medical University, No. 218, Jixi Road, Shushan District, Hefei, Anhui, 230022, PR China; William Harvey Research Institute, Faculty of Medicine and Dentistry, Queen Mary University of London, John Vane Science Centre, Charterhouse Square, London, EC1M 6BQ, UK; Department of Cardiology, Institute for Developmental and Regenerative Cardiovascular Medicine, Xinhua Hospital Affiliated to Shanghai Jiaotong University School of Medicine, Shanghai, China; William Harvey Research Institute, Faculty of Medicine and Dentistry, Queen Mary University of London, John Vane Science Centre, Charterhouse Square, London, EC1M 6BQ, UK; William Harvey Research Institute, Faculty of Medicine and Dentistry, Queen Mary University of London, John Vane Science Centre, Charterhouse Square, London, EC1M 6BQ, UK; Department of Cardiology, Institute for Developmental and Regenerative Cardiovascular Medicine, Xinhua Hospital Affiliated to Shanghai Jiaotong University School of Medicine, Shanghai, China; William Harvey Research Institute, Faculty of Medicine and Dentistry, Queen Mary University of London, John Vane Science Centre, Charterhouse Square, London, EC1M 6BQ, UK; William Harvey Research Institute, Faculty of Medicine and Dentistry, Queen Mary University of London, John Vane Science Centre, Charterhouse Square, London, EC1M 6BQ, UK; William Harvey Research Institute, Faculty of Medicine and Dentistry, Queen Mary University of London, John Vane Science Centre, Charterhouse Square, London, EC1M 6BQ, UK; Department of Cardiovascular Surgery, First Affiliated Hospital of Anhui Medical University, No. 218, Jixi Road, Shushan District, Hefei, Anhui, 230022, PR China; Department of Pharmacology, Basic Medical College, Anhui Medical University, No. 81, Meishan Road, Shushan District, Hefei, Anhui, 230032, PR China; Department of Cardiovascular Surgery, First Affiliated Hospital of Anhui Medical University, No. 218, Jixi Road, Shushan District, Hefei, Anhui, 230022, PR China; William Harvey Research Institute, Faculty of Medicine and Dentistry, Queen Mary University of London, John Vane Science Centre, Charterhouse Square, London, EC1M 6BQ, UK; Department of Cardiovascular Surgery, First Affiliated Hospital of Anhui Medical University, No. 218, Jixi Road, Shushan District, Hefei, Anhui, 230022, PR China; Department of Pharmacology, Basic Medical College, Anhui Medical University, No. 81, Meishan Road, Shushan District, Hefei, Anhui, 230032, PR China

**Keywords:** Long non-coding RNAs, Small Nucleolar RNA Host Gene 18 (SNHG18), MicroRNA, MiR-22, Adenosine Deaminase RNA Specific B1, Adenosine deaminase acting on RNA-2, Vascular smooth muscle cells, Neointima, Post-angioplasty restenosis, Cell proliferation, Cell migration

## Abstract

**Aims:**

Long non-coding RNA (LncRNA) small nucleolar RNA host gene 18 (SNHG18) has been widely implicated in cancers. However, little is known about its functional involvement in vascular diseases. Herein, we attempted to explore a role for SNHG18 in modulating vascular smooth muscle cell (VSMC) contractile phenotype and injury-induced neointima formation.

**Methods and results:**

Analysis of single-cell RNA sequencing and transcriptomic datasets showed decreased levels of SNHG18 in injured and atherosclerotic murine and human arteries, which is positively associated with VSMC contractile genes. SNHG18 was upregulated in VSMCs by TGFβ1 through transcription factors Sp1 and SMAD3. SNHG18 gene gain/loss-of-function studies revealed that VSMC contractile phenotype was positively regulated by SNHG18. Mechanistic studies showed that SNHG18 promotes a contractile VSMC phenotype by up-regulating miR-22-3p. SNHG18 up-regulates miR-22 biogenesis and miR-22-3p production by competitive binding with the A-to-I RNA editing enzyme, adenosine deaminase acting on RNA-2 (ADAR2). Surprisingly, we observed that ADAR2 inhibited miR-22 biogenesis not through increasing A-to-I editing within primary miR-22, but by interfering with the binding of microprocessor complex subunit DGCR8 to primary miR-22. Importantly, perivascular SNHG18 overexpression in the injured vessels dramatically up-regulated the expression levels of miR-22-3p and VSMC contractile genes, and prevented injury-induced neointimal hyperplasia. Such modulatory effects were reverted by miR-22-3p inhibition in the injured arteries. Finally, we observed a similar regulator role for SNHG18 in human VSMCs and a decreased expression level of both SNHG18 and miR-22-3p in diseased human arteries; and we found that the expression level of SNHG18 was positively associated with that of miR-22-3p in both healthy and diseased human arteries.

**Conclusion:**

We demonstrate that SNHG18 is a novel regulator in governing VSMC contractile phenotype and preventing injury-induced neointimal hyperplasia. Our findings have important implications for therapeutic targeting snhg18/miR-22-3p signalling in vascular diseases.

**Time of primary review: 28 days**

## 1. Introduction

Vascular smooth muscle cells (VSMCs) are a key cell component within the aortic wall and have a critical role in maintaining vascular wall integrity, vascular elasticity, blood pressure, and vascular contraction/relaxation. Upon injury or activation, the mature contractile VSMCs within the aortic tunica media undergo a dramatic adaptive process, namely contractile-synthetic phenotype switching, whereby VSMCs exhibit a marked decrease in expression of a variety of VSMC-selective marker genes alongside a rapid onset in cellular proliferation and migration.^1^ VSMC plasticity, dysfunction/dysregulation, death/senescence, clonal expansion/growth,^2^ and/or phenotypic modulation have been well-recognized as the fundamental pathological causes underlying almost all the vascular pathologies including post-angioplasty restenosis, atherosclerosis,^3–6^ aortic aneurysms and dissection,^7^ vascular calcification,^8^ arterial aging,^9^ and vascular grafting failure.^10^ Therefore, an improved understanding of the cellular and molecular mechanisms underlying the abovementioned VSMC pathologies may be beneficial for developing novel therapeutics for these vascular pathologies.

Regulatory RNAs including microRNAs (miRs),^11^ circular RNAs, and long non-coding RNAs (lncRNAs)^12,13^ have recently emerged as the master regulators in regulating VSMC pathologies such as VSMC phenotypic modulation, proliferation, and migration.^14–16^ Moreover, these regulatory RNAs have also been identified as novel therapeutic targets in cardiovascular diseases^17,18^ including atherosclerosis^19^ and neointima VSMC hyperplasia.^14,15^ As elegantly summarized across multiple reviews,^12–15,20^ although an increasing number of lncRNAs have been implicated in VSMC pathologies including SENCR (Smooth muscle and Endothelial cell-enriched migration/differentiation-associated long Non Coding RNA),^21^ SMILR (Smooth Muscle Enriched Long Noncoding RNA),^22,23^ lncPSR (lncRNA phenotype switching regulator),^24^ CARMN (cardiac mesoderm enhancer-associated noncoding RNA),^25^ MIAT (myocardial infarction-associated transcript),^26^ CARMN,^27^ and MYOSLID (MYOcardin-induced Smooth muscle LncRNA, Inducer of Differentiation),^28^ the functional implications of other lncRNAs in modulating VSMC plasticity and neointimal VSMC hyperplasia remain to be fully elucidated.

The lncRNA, small nucleolar RNA host gene 18 (abbreviated as snhg18 in mouse or SNHG18 in human), originally discovered in embryonic stem cells and provisionally named as Linc1290 by the authors,^29^ has been shown to be modulated during retinoic acid-induced embryonic stem cell differentiation.^30^ Thereafter, SNHG18 has been widely implicated in a variety of cancers. For instance, SNHG18 promotes radioresistance of glioma,^31^ enhances the growth and metastasis of glioma^32^ and non-small cell lung cancers^33^; and increased expression level of SNHG18 has been associated with poor prognosis in patients with multiple myeloma.^34^ On the other hand, other studies reported that SNHG18 functions as a tumour suppressor and potential diagnostic biomarker in hepatocellular carcinoma.^35,36^ It is increasingly understood that certain characteristics and modifiable risk factors are shared between cancer and cardiovascular diseases including post-angioplasty restenosis, and that common cellular, epi/genetic, and molecular factors underly the pathogenesis of both diseases,^37–40^ suggesting a potential role for SNHG18 in cardiovascular diseases. Importantly, we found that snhg18 was one of the most consistently up-regulated lncRNAs during VSMC differentiation from mouse adventitia stem/progenitor cells induced by transforming growth factor (TGF) beta1 (TGFβ1) in a separate stem cell study. Additionally, among the TGFβ1-mediated lncRNAs, only snhg18 has a human homology SNHG18, which prompted us to hypothesize that SNHG18 may play a role in controlling VSMC contractile phenotype and preventing injury-induced arterial remodelling. Herein, we conducted detailed biological, biochemical, and functional studies using *in vitro* and preclinical *in vivo* arterial remodelling models to gain a thorough understanding of the functional significance and therapeutic potential of SNHG18 in vascular diseases. We have provided comprehensive evidence to show that SNHG18 is an important regulator in controlling VSMC contractile-synthetic phenotype switching and preventing injury-induced neointimal hyperplasia, offering a novel therapeutic opportunity for treating post-angioplasty restenosis and other VSMC-associated diseases.

## 2. Methods

### 2.1 Animal experiments, anaesthesia, and euthanasia

All the animal procedures were approved by Queen Mary University of London ethics review board (PPL number: PP5521236), which were conducted according to the Animals (Scientific Procedures) Act of 1986 (United Kingdom) and conform to the guidelines from Directive 2010/63/EU of the European Parliament on the protection of animals used for scientific purposes or the NIH guidelines (Guide for the care and use of laboratory animals). For mouse femoral artery (FA) denudation injury and local gene transfer, anaesthesia was induced using 100% O_2_/4% isoflurane, and was maintained throughout the procedure by the administration of 100% O_2_/2% isoflurane. At the end of protocol, all mice were euthanized by placing them under deep anaesthesia with 100% O_2_/5% isoflurane, followed by decapitation.

### 2.2 Mouse femoral artery denudation injury and local gene transfer

Eight-week-old male C57BL/6 mice were anesthetized and subjected to surgical procedure as described previously.^41–50^ Briefly, a femoral arteriotomy via a groin incision was carried out aseptically with insertion of angioplasty guide-wire (0.25 mm, Hi-Torque Winn 200T guide-wire, Stock Number: 1012474, Abbott Laboratories, IL, USA) to level of aortic bifurcation for five times. After endothelium denudation, the injured arteries were randomly embedded with 100 µL of 30% pluronic gel containing 2.0 × 10^6^ lentiviral particles (Lenti-GFP [green fluorescent protein] or Lenti-snhg18) and/or 2.5 nmol miRNA inhibitor (locked nucleic acid (LNA)-SCR (scramble) or LNA-miR-22) or AgomiR (Cel-miR-67 or AgomiR-22) for local gene/miRNA transfer as described in our previous studies.^41,46–50^ Similar to our previous study,^48^ LNA modified antisense (LNA-miR-22) and scrambled control oligonucleotide (Scrambled LNA) were purchased from EXIQON Diagnostics Inc. (Copenhagen, Denmark) with their corresponding sequences −5′- CAGTTCTTCAACTGGCAGCT-3′ and −5′-ACGTCTATACGCCCA-3′. Chemically modified and cholesterol-conjugated miRNA AgomiRs were purchased from RiboBio (Guangzhou RiboBio Co., Ltd., China). Of note, Cel-miR-67 is commonly used as a negative control in miRNA research due to its minimal sequence homology with mouse, rat, and human miRNAs.

### 2.3 Analysis of human femoral arteries

Healthy and diseased human normal femoral arteries were collected from donor free from peripheral artery disease or patients with peripheral arterial diseases undergoing leg amputation at the First Affiliated Hospital of Zhejiang University (China), respectively, between July 2014 and June 2017 as described in our previous study.^48^ All studies were approved by the Research Ethics Committees of the First Affiliated Hospital of Zhejiang University (2013/150), and all experiments were conducted according to the principles expressed in the Declaration of Helsinki. Total RNA was extracted from human arterial specimens and subjected to a standard real-time quantitative PCR (RT-qPCR) analysis to examine respective mRNA/miRNA expression as described previously.^42,43,46–48,51^

### 2.4 Statistical analysis

Results are presented as mean ± standard error of the mean (SEM). Statistical analysis was performed using Graphpad Prism-8.3. Shapiro–Wilk normality test was used to check the normality of the data, and all the data passed the test. Two-tailed unpaired student’s *t*-test was used for comparisons between two groups, or one-/two-way analysis of variance (ANOVA) with a post-hoc test of Tukey’s analysis was applied when more than two groups were compared. *P* < 0.05 was considered statistically significant.

### 2.5 Additional materials and methods

An additional detailed description of materials and methods is provided in the online-only Supplementary material online, *Data.*

## 3. Results

### 3.1 Snhg18 is tightly regulated in diseased murine and human arteries as well as during VSMC contractile-synthetic phenotype switching

**Figure 1:**
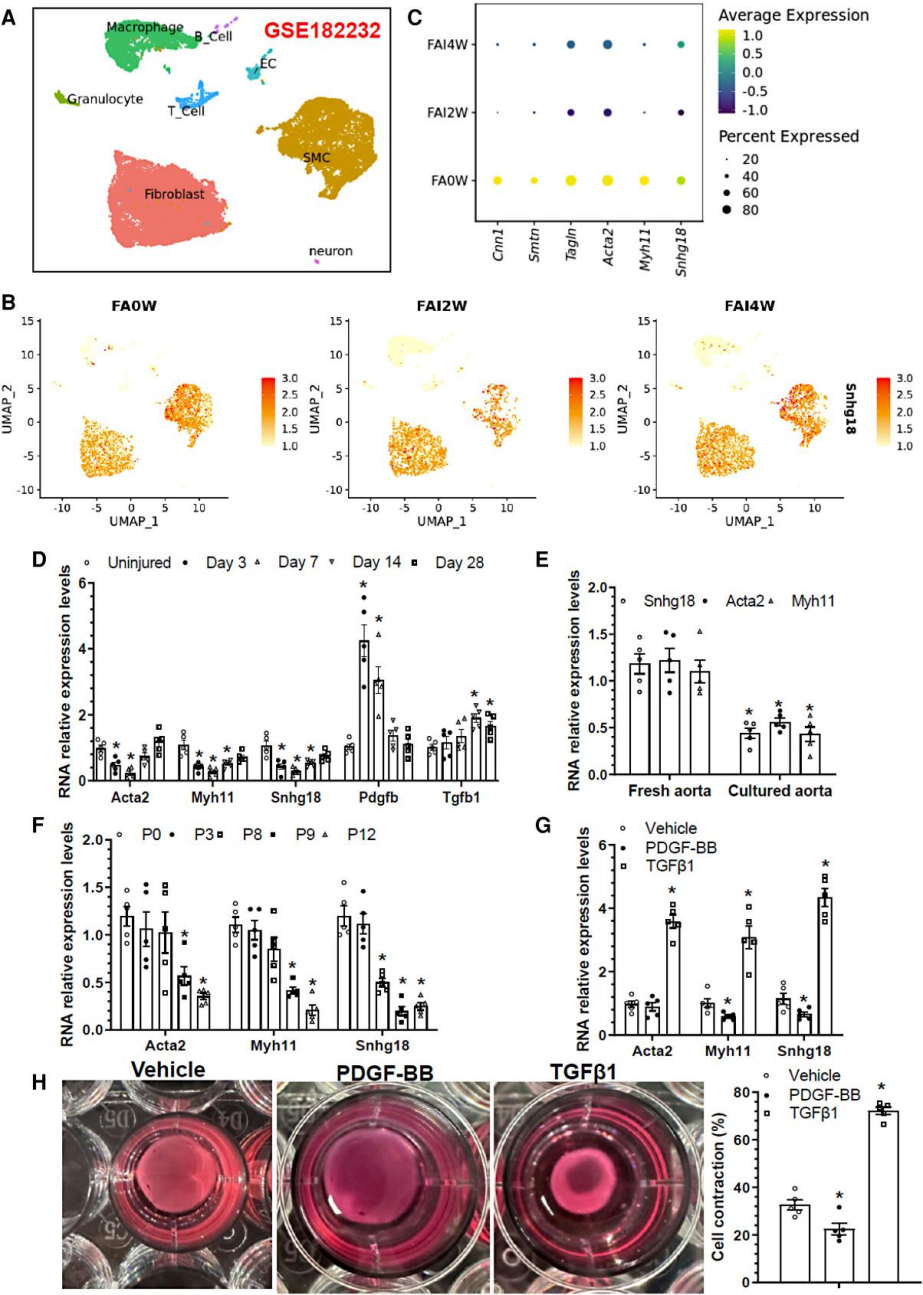
Snhg18 is tightly regulated in diseased murine and human arteries as well as during VSMC contractile-synthetic phenotype switching. (*A–C*) scRNA-Seq analysis of all cells from sham (FA0W), 2-week (FAI2W), and 4-week (FAI4W) guide-wire injured mouse femoral arteries (GSE182232). (*A*) Visualization of unsupervised clustering of the aortic cells using Uniform Manifold Approximation and Projection plot. (*B*) Distribution and expression of snhg18 gene across all aortic cell populations. (*C*) Dot plot showing average scaled expression levels of VSMC-specific genes and snhg18 in normal and injured arteries. (*D*) Gene expression profiles in injured arteries. Total RNA was collected from the uninjured and injured femoral arteries (days 3, 7, 14, and 28 post-injury), and subjected to RT-qPCR analyses. (*E*) Suppression of snhg18 in the *ex vivo* cultured thoracic aortas. Total RNA was extracted from the freshly isolated murine thoracic aortas and the aortas were cultured in Dulbecco's Modified Eagle Medium containing 20% serum for 3 days, and subjected to RT-qPCR analysis. Data in (D) and (E) represent the mean ± SEM (*n* = 5), where three injured femoral arteries were pooled as one experiment. *P < 0.05 [vs. uninjured (*D*, one-way ANOVA with a post-hoc test of Tukey’s analysis], or fresh aorta (*E*, unpaired *t*-test)]. (*F*) RT-qPCR analysis of VSMC genes and snhg18 expression levels in *in vitro* cultured VSMCs. Total RNA was harvested from freshly cultured VSMCs (cultured until day 7 and then split, designated P0), and VSMCs with the indicated passage number (P3, P8, P9, or P12), followed by RT-qPCR analysis. (*G*) Snhg18 was down-regulated by PDGF-BB, but up-regulated by TGFβ1 in cultured VSMCs. VSMCs were subjected to serum starvation for 24 h, followed by incubation with PDGF-BB (10 ng/mL) or TGFβ1 (5 ng/mL), respectively, for additional 24 h. Total RNA was extracted and subjected to RT-qPCR analysis. (*H*) Collagen-based VSMC contraction assay. Serum-starved primary murine VSMCs were incubated with vehicle control, PDGF-BB (10 ng/mL), or TGFβ1 (5 ng/mL) for additional 24 h, followed by VSMC contraction assay. Representative images (left) and percentage (%) of VSMC contraction were presented here. Data in (*F–H*) represent mean ± SEM (*n* = 5). *P < 0.05 [vs. P0 in (*F*) or Vehicle in (*G–H*) (one-way ANOVA with a post-hoc test of Tukey’s analysis)].

To examine if snhg18 expression is modulated during injury-induced arterial remodelling, we first reprocessed and re-analysed single-cell RNA sequencing (scRNA-seq) dataset (GSE182232) generated in a previous study, which was designed to study the cellular compositions in normal (FA0W) and injured (FAI2W and FAI4W) femoral arteries.^52^ Data showed that there were eight main cell types within murine aorta (VSMCs, fibroblasts, endothelial cells, macrophages, granulocytes, neuron, and B- and T-cells) (*Figure 1A*), and that snhg18 was mainly expressed in VSMCs and fibroblasts, with a much higher expression level in VSMCs (*Figure 1B*). Importantly, compared to control (FA0W), snhg18, along with VSMC contractile marker genes (Cnn1, Smtn, Tagln, Acta2, and Myh11), were decreased in injured arteries at 2 weeks post-injury (FAI2W), and their expressions were partially recovered at later stage of injury (FAI4W, 4 weeks post-injury) (*Figure 1C*). Further analysis showed that snhg18 expression had positive correlation with VSMC contractile markers in injured arteries (see Supplementary material online, *Figure S1A*). Moreover, targeted analysis of another scRNA-seq dataset (GSE155513) which was designed to reveal the trajectories of VSMC trans-differentiation during atherosclerosis^53^ showed that snhg18 was significantly and gradually decreased in all cells and VSMCs in atherosclerotic arteries in low-density lipoprotein receptor knockout (LDLR^−/−^) mice over 26 weeks of high-fat diet feeding, with a similar trend to VSMC contractile marker gene expression (see Supplementary material online, *Figure S1B*), inferring a functional role for snhg18 in controlling VSMC contractile phenotype in both injured and atherosclerotic arteries. Furthermore, we also downloaded and re-analysed a microarray dataset (GSE100927) containing transcriptomic data of atherosclerotic and healthy arteries from different human arterial beds,^54^ and found that SNHG18 was decreased in atherosclerotic arteries from all three vascular beds (carotid/femoral/infrapopliteal artery: CA/FA/IPA) (see Supplementary material online, *Figure S1C*). Finally, targeted analysis of RNA-seq dataset (GSE120521) which was designed to assess transcriptomic differences between stable and unstable human carotid plaque^23^ revealed a trend of decreased expression level of SNHG18 in unstable human plaques (see Supplementary material online, *Figure S1D*). Taken together, these data collectively suggest a potential role for SNHG18 in VSMC contractile-synthetic phenotype switching in the context of injury-induced arterial remodelling and atherogenesis.

Multiple *in vivo*, *ex vivo,* and *in vitro* VSMC phenotype switching models were conducted as described in our previous study^48^ to further confirm snhg18 gene modulation during VSMC contractile-synthetic phenotype switching. Wire/balloon-induced arterial injury in mice has been extensively used to mimic coronary angioplasty in humans. This procedure promotes VSMC contractile-synthetic phenotype switching as evidenced by increased VSMC proliferation and migration but a steady decline in the expression of VSMC differentiation markers in injured murine arteries.^55,56^ By using such a well-established *in vivo* VSMC phenotype switching model, we first found that snhg18, along with VSMC genes (Acta2 and myh11), were significantly decreased in the early stages (3, 7, and 14 days) of injured femoral arteries, which were partially or completely recovered at a late stage (28 days post-injury) (*Figure 1D*). Additionally, pdgfb gene expression was up-regulated and peaked at day 3, while tgfb1 gene expression was gradually increased upon injury (*Figure 1D*). Snhg18 expression in normal and injured arteries was further confirmed by fluorescence *in situ* hybridization (FISH) (see Supplementary material online, *Figure S2*). Moreover, studies from our own^48^ and others^57^ show that VSMCs in the cultured aortic tissues undergo a dramatic switch towards a synthetic proliferative phenotype. Indeed, RT-qPCR data showed a significant decrease in expression levels of snhg18 and VSMC contractile genes in *ex vivo* explanted cultured thoracic aortic tissues (*Figure 1E*). Similar to our previous findings,^48^ we found that *in vitro* cultured VSMCs at early passages (up to passage 8, P8) displayed a comparable level of VSMC contractile markers expression, but these VSMC contractile genes were dramatically down-regulated in extended cultured VSMCs (between passage 9 and 12) (*Figure 1F*), indicate of VSMC contractile-synthetic phenotype switching under prolonged *in vitro* culture. Accordingly, VSMCs between passages 3–8 were used in this study. Interestingly, we observed a similar expression trend for snhg18 in the cultured VSMCs (*Figure 1F*). Finally, the expression levels of snhg18 together with VSMC genes in cultured and serum-starved VSMCs were significantly down-regulated by platelet-derived growth factor BB (PDGF-BB) stimulation, a condition known to inhibit VSMC contractile protein expression and promote VSMC proliferation and migration,^58^ but dramatically up-regulated by transforming growth factor β1 (TGFβ1) treatment, a condition known to induce a contractile VSMC phenotype by increasing VSMC marker gene expressions^59,60^ (*Figure 1G*). Indeed, data from collagen-based cell contraction assay showed that PDGF-BB inhibited, but TGFβ1 increased VSMC contraction (*Figure 1H*). These data have collectively shown that snhg18 is tightly regulated during VSMC contractile-synthetic phenotype switching *in vivo*, *ex vivo*, and *in vitro*.

### 3.2 TGFβ1 upregulates snhg18 in VSMCs via transcription factors Sp1 and SMAD3

**Figure 2:**
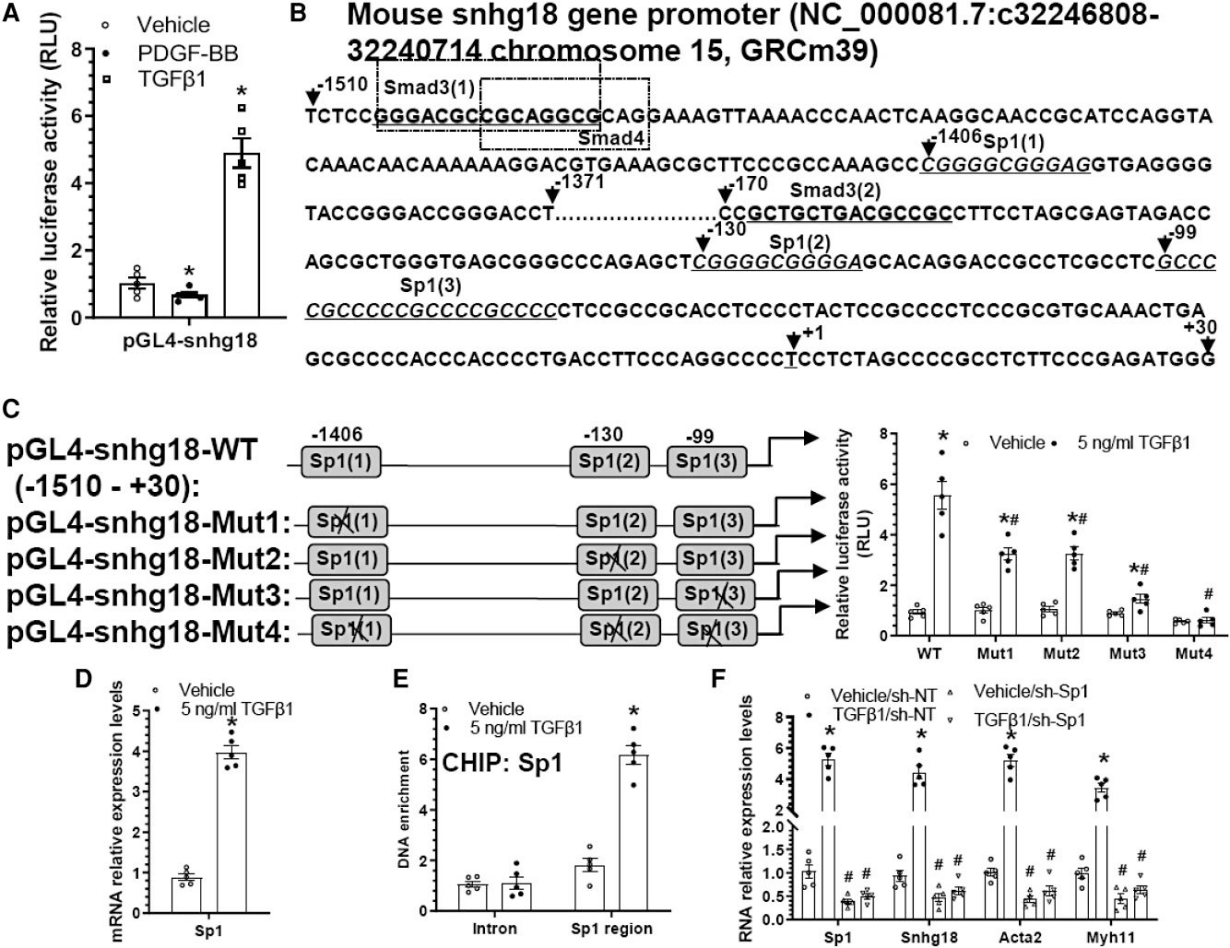
TGFβ1 upregulates snhg18 and VSMC gene expression by modulating transcription factor Sp1. (*A*) PDGF-BB significantly decreases, whereas TGFβ1 significantly increases snhg18 gene promoter activity. VSMCs transfected with snhg18 gene promoter (pGL4-snhg18) were subjected to serum starvation for 24 h, followed by incubation with control medium (vehicle), PDGF-BB (10 ng/mL), or TGFβ1 (5 ng/mL) for 24 h. Cell lysates were harvested and analysed using the luciferase activity assay. (*B*) Schematic illustration of DNA sequence for mouse snhg18 gene promoter and potential binding sites for transcription factor Sp1, and SMAD3/4. + 1 indicates translation initiation site. (*C*) Luciferase activity analysis of mouse snhg18 gene promoters. Left panel, schematic diagram of wild-type (pGL4-snhg18-WT) and mutated (pGL4-snhg18-MUT1–4) snhg18 gene promoter reporters. Right panel, luciferase activity assays with the indicated reporters. (*D*) TGFβ1 upregulated Sp1 in VSMCs. (*E*) CHIP assay showing TGFβ1 promotes Sp1 binging to snhg18 gene promoter. (*F*) Upregulation of Sp1 is essential for TGFβ1-mediated snhg18 gene regulation and VSMC gene expression. VSMCs infected with control (sh-NT) or Sp1 gene-specific (sh-Sp1) shRNA lentivirus were subjected to serum starvation for 24 h, followed by TGFβ1 (5 ng/mL) treatment for further 24 h. Total RNA was extracted and subjected to RT-qPCR analysis. Data and error bars represent the mean ± SEM (*n* = 5). *P < 0.05 (vs. vehicle in *A*, *C*–*F*); ^#^*P* < 0.05 (sh-Sp1 vs. sh-NT in *F*) (One- or two-way ANOVA with a post-hoc test of Tukey’s analysis in *A* and *C*/*F*, respectively; unpaired *t*-test in *D* and *E*).

In the above section, we have shown that TGFβ1 and PDGF-BB up-regulate and down-regulate snhg18 gene expression, respectively. To further distinguish if snhg18 is regulated by TGFβ1 and PDGF-BB through a transcriptional or post-transcriptional mechanism in VSMCs, we first generated a snhg18 gene promoter-reporter and conducted luciferase activity assay. Luciferase activity assay data showed that while PDGF-BB inhibited snhg18 gene promoter activity, it was significantly increased by TGFβ1 (*Figure 2A*), inferring that both PDGF-BB and TGFβ1 regulate snhg18 gene expression at a transcriptional level. After closely scrutinizing the promoter sequence of the murine snhg18 gene, we found multiple binding sites for the transcription factor Sp1 existed within the snhg18 gene promoter (*Figure 2B*). Indeed, data from luciferase activity assays using snhg18 gene reporters containing either wild type (WT) or Sp1 binding site mutant variants showed that compared to WT reporter the luciferase activity was significantly decreased in cells transfected with reporters harbouring individual Sp1 binding site mutations (pGL4-snhg18-Mut1/2/3). Importantly, snhg18 gene promoter activity induced by TGFβ1 was completely abolished when all three Sp1 binding sites were mutated (pGL4-snhg18-Mut4) (*Figure 2C*), confirming a critical role for these Sp1 binding sites in snhg18 gene regulation by TGFβ1. Interestingly, the reporter with a mutation in all three Sp1 binding sites produced the least luciferase activity in cells treated with or without TGFβ1 among all the experimental groups (*Figure 2C*), indicating a regulator role for Sp1 in regulating snhg18 expression at a basal level. Moreover, RT-qPCR analysis showed an increase in Sp1 gene expression in VSMCs treated with TGFβ1 (*Figure 2D*). Furthermore, chromatin immunoprecipitation (CHIP) assays revealed a direct binding of Sp1 to the snhg18 gene promoter, which was significantly increased by TGFβ1 (*Figure 2E*). Finally, we found that Sp1 knockdown significantly inhibited snhg18 gene expression in VSMCs treated with or without TGFβ1 (*Figure 2F*). A similar trend was observed with VSMC marker genes (*Figure 2F*), indicating that Sp1 activation is required for TGFβ1-induced snhg18 and VSMC marker gene expression. As expected, Sp1 gene expression was also decreased in injured arteries at early stage, but returned to a normal level at later stage (see Supplementary material online, *Figure S3A*). Immunostaining data further confirmed decreased levels of Sp1 and smooth muscle alpha actin (SMA) in injured arteries (see Supplementary material online, *Figure S3B* and *C*), inferring a regulatory role for Sp1 in snhg18 expression in injured arteries. Altogether, these data confirm an important regulatory role for Sp1 in TGFβ1-induced snhg18 and VSMC gene expression.

It is widely known that small mothers against decapentaplegic homolog (SMAD) proteins, particularly the receptor-regulated SMADs (SMAD2/3) and the common partner SMAD (SMAD4|), are the principal intracellular mediators of TGFβ superfamily signalling,^61^ which prompted us to study if TGFβ1 upregulates snhg18 expression also through these SMADs. Indeed, two SMAD3/4 binding sites within the snhg18 gene promoter were predicted with high scores (*Figure 2B* and Supplementary material online, *Figure S4A*). Accordingly, multiple reporters containing snhg18 gene promoter sequences with (WT) or without SMAD binding site(s) were generated as shown in Supplementary material online, *Figure S4B* (left). Data from luciferase activity assays showed a decreased gene promoter activity when the SMAD3/4 binding site was deleted (see Supplementary material online, *Figure S4B*, right), confirming a functional role for these SMAD3/4 binding sites in snhg18 gene regulation by TGFβ1.

### 3.3 Snhg18 is predominantly enriched in the VSMC nucleus

It has been well-known that the cellular locations of lncRNAs determine their potential cellular functions and molecular actions in normal physiological development and disease pathogenesis.^62–64^ We first extracted nuclear and cytoplasmic RNAs from serum-starved VSMCs and examined the expression levels of snhg18 in different RNA fractions. As expected, RT-qPCR data showed that while glyceraldehyde-3-phosphate dehydrogenase (GAPDH) was mainly enriched in cytosol, U6 small nuclear RNA (U6) was mainly expressed in nucleus (see Supplementary material online, *Figure S5A*). Similar to U6, snhg18 was predominantly enriched in nuclear RNA fraction (see Supplementary material online, *Figure S5A*). The nuclear location of snhg18 in serum-starved VSMCs (see Supplementary material online, *Figure S5B*) and injured arteries (see Supplementary material online, *Figure S2*) was further confirmed by FISH assay.

### 3.4 Snhg18 controls VSMC contractile phenotype

**Figure 3:**
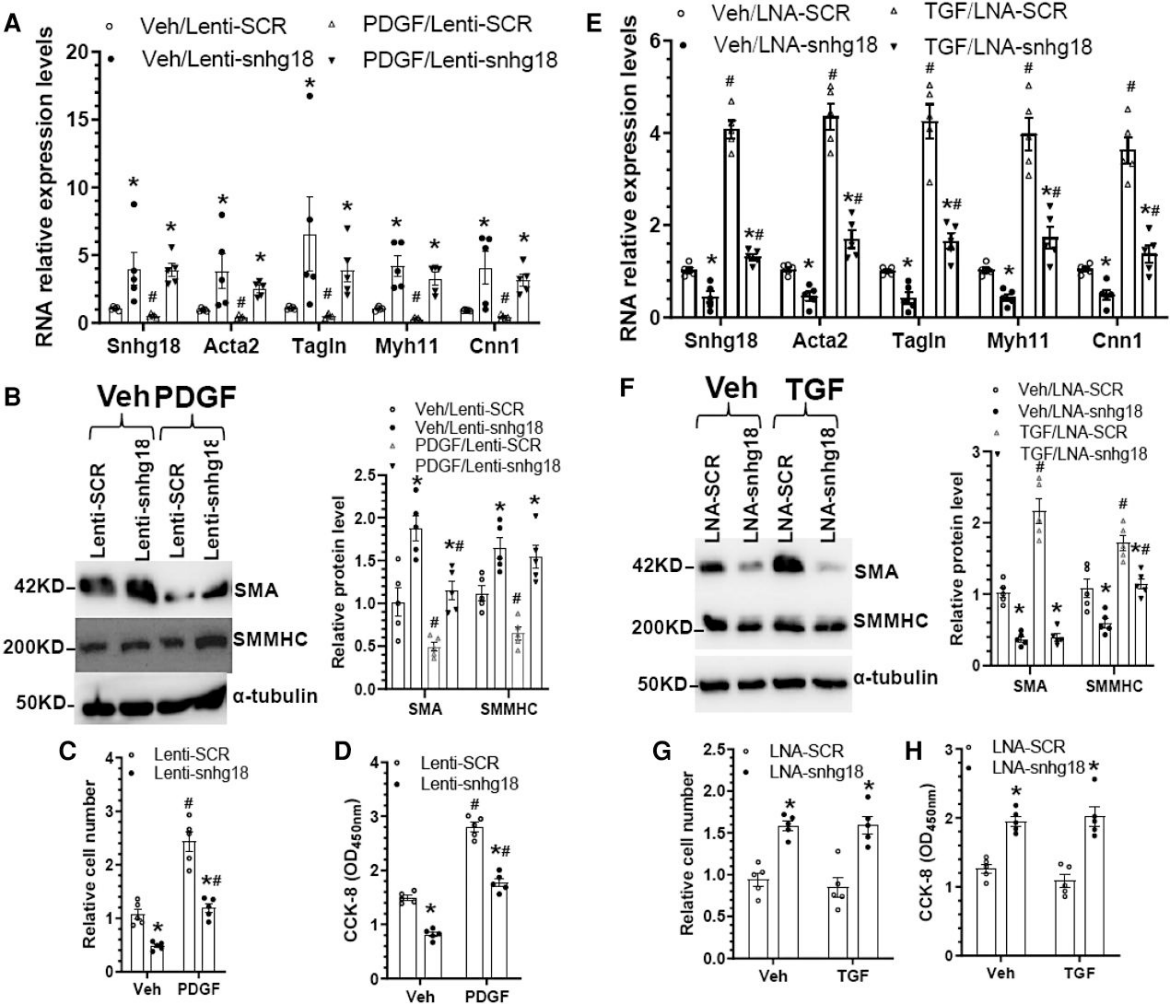
Snhg18 modulates VSMC phenotype. (*A–D*) Snhg18 overexpression upregulated VSMC gene expression but decreased VSMC proliferation. VSMCs infected with control (lenti-SCR) or snhg18 overexpression (lent-snhg18) lentivirus were subjected to serum starvation for 24 h. After that, cells were treated with vehicle control (Veh) or PDGF-BB (10 ng/mL) for further 24 h, followed by RT-qPCR (*A*), Western blot (*B*), cell count (*C*), and cell counting kit (CCK)-8 (*D*) assays, respectively. (*E–H*) Snhg18 knockdown down-regulated VSMC gene expression but promoted VSMC proliferation. VSMCs transfected with control (LNA-SCR) or snhg18 gene-specific (LNA-snhg18) antisense locked nucleotide acid (LNA) GapmeRs were subjected to serum starvation for 24 h. After that, cells were treated with vehicle control (Veh) or TGFβ1 (5 ng/mL, transforming growth factor (TGF)) for further 24 h, followed by RT-qPCR (*E*), Western blot (*F*), cell count (*G*), and CCK-8 (*H*) analysis, respectively. The data presented here are representatives (left panel in B and *F*) or mean ± SEM (data and error bars in other panels) of five (*n* = 5) independent experiments. *P < 0.05 (vs. Lenti-SCR in A–D or LNA-SCR in E–H), #*P* < 0.05 (PDGF vs. Veh in *A*–*D* or TGF Vs Veh in E–H) (two-way ANOVA with a post-hoc test of Tukey’s analysis).

Our above data showed that the expression level of snhg18 is closely modulated during VSMC phenotype switching. We speculated that snhg18 may play a functional role in controlling VSMC contractile phenotype. To determine whether snhg18 is essential and sufficient for maintaining VSMC contractile phenotype, snhg18 gene gain/loss-of-function studies were conducted in VSMCs using a snhg18 overexpression lentiviral vector (Lenti-snhg18) and a snhg18 antisense locked nucleotide acid (LNA) GapmeRs (LNA-snhg18), respectively, followed by various analyses. It is worth noting that PDGF-BB or TGFβ1 treatment was specifically included in the snhg18 gene gain- or loss-of-function experiments to better illustrate a functional role of snhg18 in VSMC switching from contractile to synthetic phenotype, since we found that snhg18 and VSMC contractile marker genes were significantly down-regulated and up-regulated by PDGF-BB and TGFβ1, respectively (*Figure 1D*). Indeed, RT-qPCR data showed that snhg18 overexpression significantly increased expression levels of all four VSMC contractile genes (Acta2, Tagln, Myh11, and Cnn1) in serum-starved VSMCs treated with control vehicle and PDGF-BB (*Figure 3A*). Importantly, we found that while these VSMC contractile genes were down-regulated by PDGF-BB, such inhibitor effects were partially or fully rescued by snhg18 overexpression (*Figure 3A*). These observations were further confirmed at the protein level (*Figure 3B*). Functionally, snhg18 overexpression significantly inhibited VSMC proliferation (*Figure 3C and D*) and migration (*Figure S6A and B*) in the absence or presence of PDGF-BB treatment. Conversely, expression of VSMC contractile marker genes was significantly down-regulated by snhg18 knockdown in serum-starved VSMCs incubated with or without TGFβ1 (*Figure 3E and F*). Meanwhile, we observed that snhg18 knockdown significantly increased VSMC proliferation (*Figure 3G and H*) and migration (see Supplementary material online, *Figure S6C* and *D*). These data have collectively shown that snhg18 is a key regulator for controlling VSMC contractile-synthetic phenotype switching. Moreover, it is important to note that a similar regulatory effect of snhg18 gene manipulation on VSMC contractile marker gene expression, proliferation, and migration was observed in serum-starved VSMCs treated with or without stimuli (PDGF-BB or TGFβ1), suggesting that snhg18-mediated VSMC contractile gene expression, proliferation and migration is stimuli–independent.

### 3.5 Snhg18 maintains VSMC contractile phenotype by upregulating miR-22-3p

**Figure 4:**
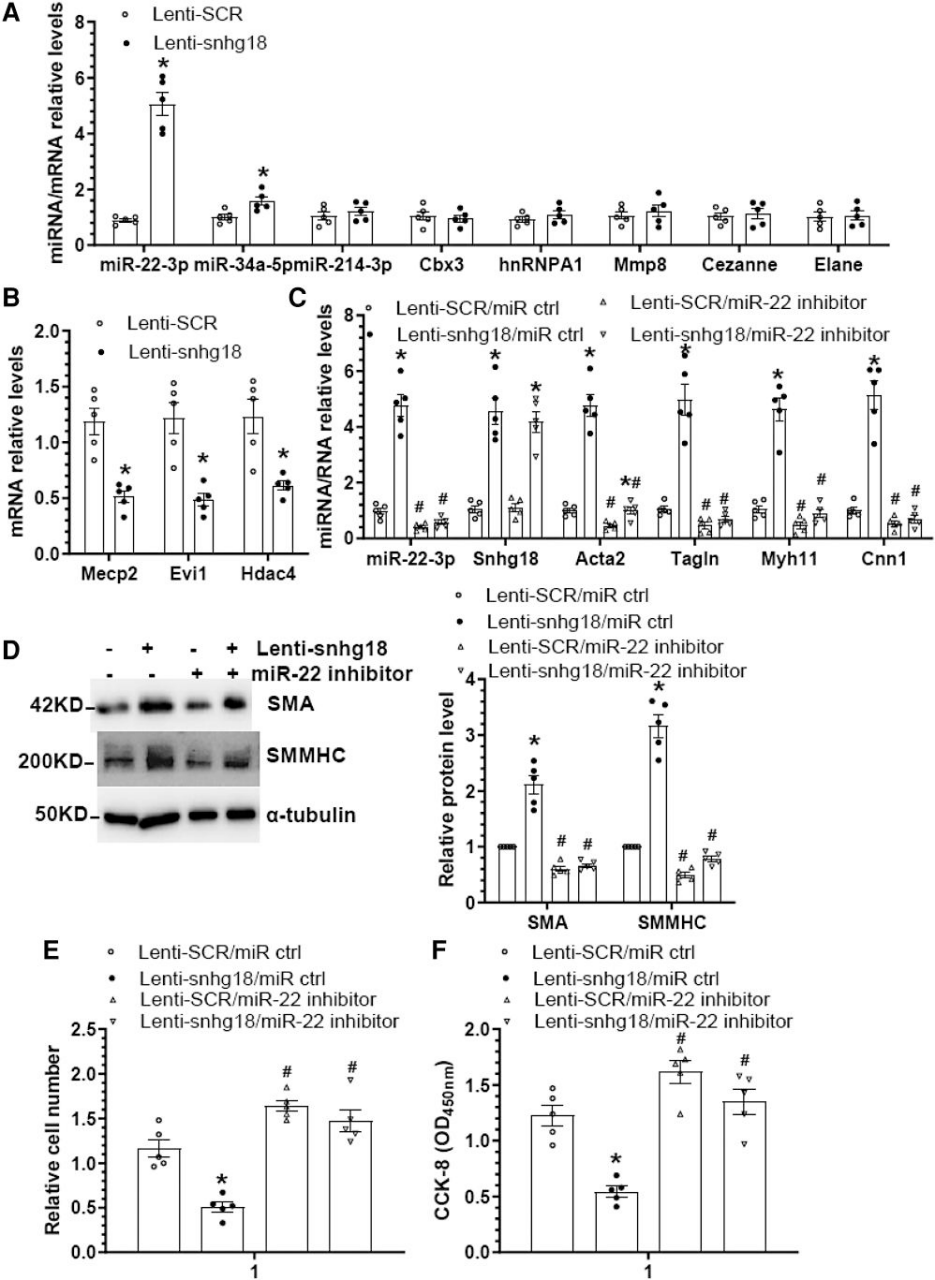
Snhg18 modulates VSMC phenotype by upregulating miR-22-3p. (*A* and *B*) Snhg18 overexpression increased miR-22-3p and decreased its target genes. VSMCs were infected with control (Lenti-SCR) or snhg18 overexpression (Lent-snhg18) lentivirus and subjected to serum starvation for 24 h. Total RNA was extracted and subjected to RT-qPCR analysis. (*C–F*) The modulatory effects of snhg18 overexpression on VSMC marker gene expression and proliferation were blunted by miR-22-3p inhibition. VSMCs infected with Lenti-SCR or Lenti-snhg18 were transfected with a control (miR Ctrl) or miR-22-3p inhibitor, respectively. After then, cells were subjected to serum starvation for 24 h, followed by RT-qPCR (*C*), Western blot (*D*), cell count (*E*), and CCK-8 (*F*) analysis, respectively. The data presented here are representatives (left panel in *D*) or mean ± SEM (data and error bars in other panels) of five (*n* = 5) independent experiments. *P < 0.05 (vs. Lenti-SCR), #*P* < 0.05 (miR-22 inhibitor vs. miR Ctrl) (unpaired *t*-test in *A*/*B*; two-way ANOVA with a post-hoc test of Tukey’s analysis in C–E).

We have previously identified miR-22-3p,^48^ miR-34a-5p,^50^ miR-214-3p,^49^ chromobox homologue 3 (Cbx3),^46^ heterogeneous nuclear ribonucleoprotein A1 (hnRNPA1),^41^ matrix metallopeptidase 8 (Mmp8),^44^ Cezanne,^47^ and neutrophil elastase (Elane)^42^ as key modulators in controlling VSMC contractile/synthetic phenotypes, proliferation and migration, as well as injury-induced arterial remodelling. We, therefore, considered whether snhg18 controls VSMC contractile/synthetic phenotypes and functions by regulating these genes. To answer this question, we first examined if expressions of these genes were impacted by snhg18, and found that snhg18 overexpression significantly up-regulated miR-22-3p and to a lesser extent, miR-34a-5p expression, but had no apparent effect on other gene expression (*Figure 4A*), indicating a role for snhg18 in miR-22-3p regulation. Importantly, all three target genes of miR-22-3p in the context of VSMC functions were dramatically down-regulated by snhg18 (*Figure 4B*), further supporting a regulatory role of snhg18 in miR-22-3p signalling. To further explore a potential role of miR-22-3p in snhg18-mediated VSMC contractile marker genes expression, proliferation, and migration, serum-starved VSMCs were co-transfected with a snhg18 overexpression vector and a miR-22-3p inhibitor, followed by various analysis. Data from RT-qPCR and western blot assays showed that while VSMC contractile marker genes were significantly up-regulated and down-regulated by snhg18 overexpression and miR-22-3p inhibition, respectively, the promotive effects of snhg18 overexpression were abolished by a miR-22-3p inhibitor (*Figure 4C and D*). As expected, while snhg18 overexpression and miR-22-3p inhibition alone decreased and increased VSMC proliferation, respectively, the modulatory effects of snhg18 overexpression disappeared in the presence of miR-22-3p inhibitor (*Figure 4E and F*). A similar phenomenon was observed in PDGF-BB-induced VSMC migration (see Supplementary material online, *Figure S7A* and *B*). Functionally, we found that while VSMC contraction was significantly increased and decreased by snhg18 overexpression and miR-22-3p inhibition, respectively, the promotive effects of snhg18 overexpression on VSMC contraction were blunted by miR-22-3p inhibition (see Supplementary material online, *Figure S7C* and *D*). Importantly, similar modulatory effects of snhg18/miR-22-3p co-transfection on VSMC contractile genes expression and proliferation were observed in VSMCs treated with TGFβ1 (see Supplementary material online, *Figure S8*). Altogether, the above data confirm that Snhg18 regulates VSMC contractile/synthetic phenotype switching through upregulation of miR-22-3p.

### 3.6 Snhg18 regulates miR-22 biogenesis and miR-22-3p production by competitive binding with ADAR2

**Figure 5:**
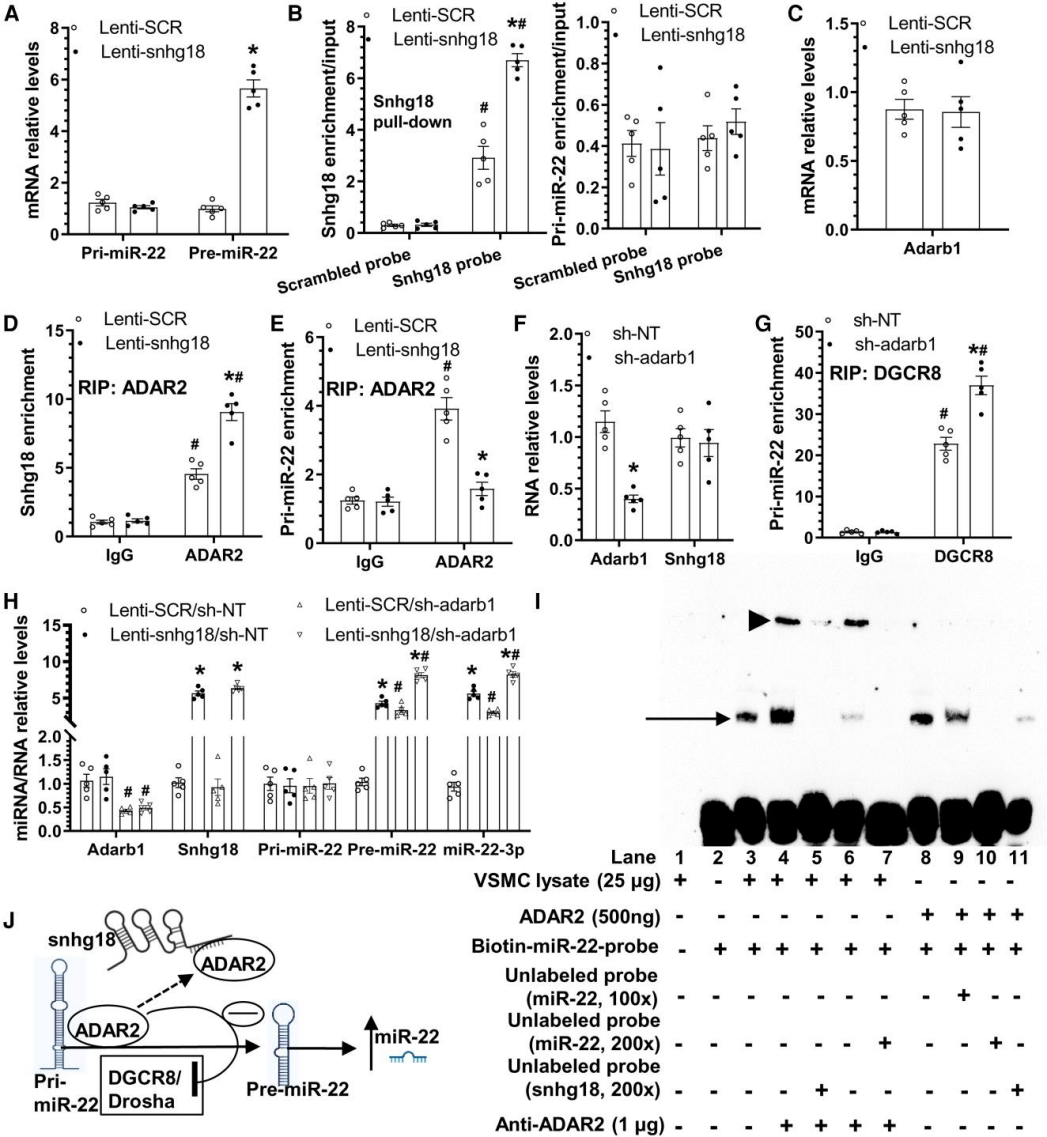
Snhg18 regulates miR-22 biogenesis and miR-22-3p production by competitive binding with ADAR2. (*A*) Snhg18 overexpression upregulated precursor (pre-miR-22) but not the primary (Pri-miR-22) miR-22 expression levels in VSMCs. (*B*) Snhg18 RNA pull-down assay. VSMCs were infected with control (Lenti-SCR) or snhg18 overexpression (Lent-snhg18) lentivirus and subjected to serum starvation for 24 h, followed by RNA pull-down using a biotinylated scrambled or snhg18-specific oligonucleotide probe. Purified RNA was subjected to RT-qPCR analysis to detect snhg18 and pri-miR-22, respectively. (*C*) RT-qPCR analysis of Adarb1 gene expression. (*D*, *E*) RNA immunoprecipitation (RIP) assay with IgG control or ADAR2 antibody. (*F*, *G*) Adarb1 knockdown increased DGCR8 binding to pri-miR-22. VSMCs were infected with non-target (NT) (sh-NT) or Adarb1 gene-specific (sh-Adarb1) shRNA lentivirus and subjected to serum starvation for 24 h, followed by RT-qPCR analysis (*F*) and RIP assay using a IgG control or DGCR8 antibody (*G*), respectively. (*H*) Pre-miR-22 and miR-22-3p were synergistically increased by snhg18 overexpression and adarb1 gene knockdown. VSMCs were co-infected with a control and gene-specific overexpression/knockdown vector as indicated in the figure and subjected to serum starvation for 24 h, followed by RT-qPCR analysis. Data and error bars in (*A–H*) represent mean ± SEM of five (*n* = 5) independent experiments. *P < 0.05 (vs. Lenti-SCR in A–E/H, or sh-NT in F/G), #*P* < 0.05 (vs. scrambled probe in B, IgG in D/E/G, or sh-adarb1 vs. sh-NT in *H*) (Unpaired *t*-test in A/C; two-way ANOVA with a post-hoc test of Tukey’s analysis in B and D–H). (*I*) RNA electrophoretic mobility shift assay. Representative image from three experiments (*n* = 3) was presented here. Arrowhead and arrow indicate super-shifting and shifting band, respectively. (*J*) Schematic illustration of the model of action of snhg18 in promoting miR-22-3p production.

To determine whether snhg18 regulates miR-22-3p through modulation of miRNA transcriptional or regulation of miRNA biogenesis, we first measured the expression levels of primary (Pri-miR-22) and precursor (Pre-miR-22) miR-22 transcript in VSMCs. RT-qPCR data showed that snhg18 significantly increased Pre-miR-22, but not the Pri-miR-22 in serum-starved VSMCs (*Figure 5A*), suggesting that snhg18 regulates miR-22 precursor biogenesis from its primary transcript. A high likelihood of mouse snhg18 directly binding mmu-miR-22 was predicted by RNAhybrid (https://bibiserv.cebitec.uni-bielefeld.de/rnahybrid) with good confidence (see Supplementary material online, *Figure S9*). To confirm such a direct binding, RNA pull-down experiment using a biotinylated snhg18-specific oligonucleotide probe was conducted in serum-starved VSMCs. Compared with the scrambled RNA probe, the snhg18 transcript was significantly enriched by the snhg18 probe in control VSMCs, which was further increased by snhg18 overexpression (*Figure 5B*). However, no enrichment was observed with Pri-miR-22 transcript (*Figure 5B*), indicating that there is no direct interaction between snhg18 and pri-miR-22 in VSMCs. It is well-recognized that lncRNAs may also interact with RNA binding proteins and exert their cellular functions in this way. Therefore, we used the bioinformatics tool ATtRACT (https://attract.cnic.es/seqsearch) to predict potential RNA binding proteins of snhg18. Eighteen RNA binding proteins were predicted as likely targets of interaction with snhg18 (see Supplementary material online, *Table S2*). Among them, the A-to-I RNA editing enzyme, adenosine deaminase acting on RNA-2 (ADAR2) or adenosine deaminase RNA Specific B1 (Adarb1), caught our attention since it has been widely reported to play a critical role in controlling miRNA biogenesis and production.^65–68^ To explore a possible role for ADAR2 in snhg18-mediated miR-22 regulation, we first examined if the Adarb1 gene expression was impacted by snhg18 in VSMCs, and found that Adarb1 gene expression was not regulated by snhg18 overexpression (*Figure 5C*). However, data from our RNA immunoprecipitation (RIP) assay with ADAR2 antibody revealed a direct binding between ADAR2 and snhg18 (*Figure 5D*) or Pri-miR-22 (*Figure 5E*) RNA transcript. Interestingly, we found that while snhg18 overexpression further increased the biding of ADAR2 to snhg18, it significantly inhibited the binding between ADAR2 and Pri-miR-22, suggesting that snhg18 negatively regulates the interaction between ADAR2 and Pri-miR-22 by sequestering ADAR2 from the ADAR2/Pri-miR-22 complex. Moreover, it is widely known that the microprocessor complex subunit DGCR8 is a critical RNA binding protein in governing the biogenesis of miRNAs from their primary transcript. Data from RIP assay with the DGCR8 antibody confirmed direct binding of DGCR8 to Pri-miR-22 transcript, which was further enhanced by Adarb1 gene knockdown (*Figure 5F and G*), suggesting an inhibitory effect of ADAR2 on the interaction between DGCR8 and Pri-miR-22, and possibly on Pri-miR-22 bioprocessing to Pre-miR-22. For further confirmation, a co-infection experiment using a snhg18 overexpression and an Adarb1 gene-specific shRNA lentivirus was conducted in serum-starved VSMCs. RT-qPCR analysis data showed that snhg18 overexpression or Adarb1 knockdown alone significantly up-regulated both Pre-miR-22 and miR-22-3p, and the promotive effects of snhg18 overexpression on Pre-miR-22 and miR-22-3p were further enhanced by Adarb1 inhibition (*Figure 5H*). However, no such regulation was observed with Pri-miR-22 (*Figure 5H*), inferring an antagonistic effect of snhg18 and ADAR2 on miR-22 biogenesis and production.

It is well-known that ADAR2-mediated A-to-I editing within pri-miRNA transcripts is one of the key mechanisms through which ADAR2 regulates miRNA biogenesis and production.^67,68^ We wondered if a similar mechanism was responsible for ADAR2-mediated miR-22-3p generation. Data from an Endonuclease V immunoprecipitation enrichment (EndoVIPER) analysis showed that the A-to-I RNA editing within Pri-miR-142 was significantly increased by overexpression of the wild type (Lent-adarb1), but not by the catalytically inactive adarb1 variant (Lenti-adarb1 (E396A)),^69,70^ which is consistent with previously reported findings.^65,66^ Surprisingly, we observed a low level of A-to-I RNA editing within Pri-miR-22 (see Supplementary material online, *Figure S10A*). As expected, miR-142 was dramatically suppressed by ADAR2, while such an inhibitory effect disappeared in VSMCs infected with Lenti-adarb1 (E396A) (see Supplementary material online, *Figure S10B*), confirming ADAR2-mediated A-to-I RNA editing within Pri-miR-142 is responsible for ADAR2-mediated miR-142 suppression. In contrast, both Pre-miR-22 and miR-22-3p were significantly inhibited in VSMCs infected with either Lenti-Adarb1 or Lenti-adarb1 (E396A) overexpression lentivirus (see Supplementary material online, *Figure S10B*), suggesting that ADAR2 inhibition of miR-22 generation is independent of A-to-I RNA editing.

To further confirm a competitive binding of snhg18 and Pri-mir-22 to ADAR2, RNA electrophoretic mobility shift assay (RNA-EMSA) was conducted using VSMC nuclear lysate (lanes 1–7) or mouse recombinant ADAR2 protein (lanes 8–11) (*Figure 5I*). We observed a specific shifting band (red arrow, *Figure 5I*) when ADAR2 protein was incubated with biotin-pri-miR-22 probe (lane 8), which was partially and completely inhibited by 100 × (lane 9) and 200 × (lane 10) unlabelled pri-miR-22 probe, respectively. Crucially, the direct interaction between pri-miR-22 and ADAR2 protein was almost blocked by 200 × unlabelled snhg18 probe (lane 11). A similar shifting band (red arrow) and a super-shifting band (red arrow-head, *Figure 5I*) were also observed with VSMC nuclear lysate in the absence (lane 3) or presence (lanes 4 and 6) of ADAR2 antibody, which was largely blocked by unlabelled snhg18 (lane 5) or Pri-miR-22 (lane 7) probe, respectively, confirming direct and competitive binding of snhg18 and Pri-mir-22 to ADAR2. Altogether, these data have collectively demonstrated that snhg18 promotes miR-22 biogenesis by sequestering ADAR2 from ADAR2/Pri-miR-22 complex, which allows a more efficient interaction between DGCR8 and Pri-miR-22 transcript, thereby increasing miR-22 production (*Figure 5J*).

Finally, we examined some key molecular regulatory events observed from snhg18 overexpression experiments in VSMCs treated with TGFβ1. We found that TGFβ1 treatment significantly increased Pre-miR-22, but not the Pri-miR-22 in serum-starved VSMCs (see Supplementary material online, *Figure S11A*), and that adarb1 gene expression was not affected by TGFβ1 (see Supplementary material online, *Figure S11B*). Similarly, data from RIP assays showed that TGFβ1 increased the direct binding of snhg18 to ADAR2 (see Supplementary material online, *Figure S11C*), but significantly decreased the interaction between ADAR2 and Pri-miR-22 (see Supplementary material online, *Figure S11D*). These data confirmed a similar regulatory network for miR-22 biogenesis under pathophysiological condition with TGFβ1 treatment.

### 3.7 Snhg18 reduces injury-induced neointima formation by modulating miR-22

**Figure 6:**
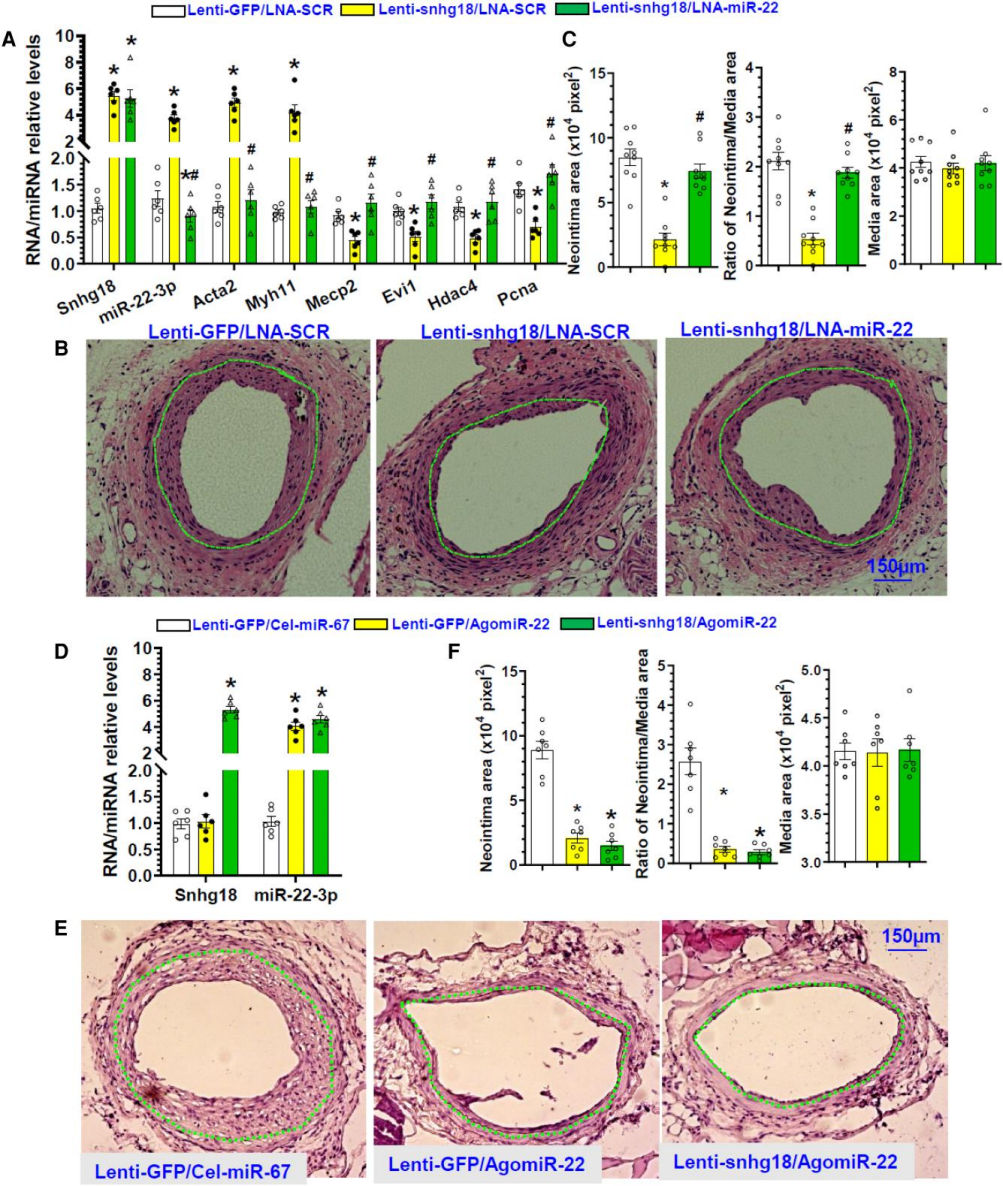
Snhg18 reduces injury-induced neointima formation by modulating miR-22. After injury, 100µL of 30% pluronic gel contained 2.0 × 10^6^ lentiviral particles (Lenti-GFP or Lenti-snhg18) and/or 2.5 nmol miRNA inhibitor (LNA-SCR or LNA-miR-22, *A*–*C*), or 2.5 nmol AgomiRs (Cel-miR-67 or AgomiR-22-3p, *D*–*F*) was immediately applied and packed around the injured vessel. Injured segments of femoral arteries collected at 7 (*A* and *D*) or 28 (*B*, *C* and *E*, *F*) days post-injury were prepared and subjected to RT-qPCR (*A* and *D*), or hematoxylin and eosin staining (*B* and *E*) analysis, respectively. Data presented here are representative images (*B* and *E*) or mean ± S.E.M from six independent experiments (*n* = 6, *A* and *D*), 9 (*B* and *C*) or 7 (*E* and *F*) mice. Notes: RNAs from three injured femoral arteries were pooled as 1 experiment in (*A* and *D*). *P < 0.05 (vs. Lenti-GFP/LNA-SCR or Lenti-GFP/Cel-miR-67), ^#^*P* < 0.05 (vs. Lenti-snhg18/LNA-SCR) (one-way ANOVA with a post-hoc test of Tukey’s analysis).

It has been well-established as well as observed in our previous studies that upon vascular injury, contractile VSMCs undergo a dramatic change and switch towards a synthetic phenotype, expressing a reduced level of VSMC contractile genes but with an increased capacity for proliferation/migration.^41–50^ Importantly, we observed a reduced expression of snhg18 in the murine femoral arteries following wire/balloon-induced injury (*Figure 1D* and Supplementary material online, *Figure S2*). Moreover, we found that miR-22-3p was significantly down-regulated in injured arteries, and locally enforced expression or inhibition of miR-22-3p decreased or increased neointimal hyperplasia in injured femoral arteries,^48^ suggesting a functional role for the snhg18/miR-22-3p signalling axis in post-angioplasty restenosis. To test this hypothesis, 100 μL of 30% pluronic gel containing a control or snhg18 overexpression lentivirus together with chemically modified and cholesterol conjugated scrambled or miR-22-3p LNA inhibitor was perivascularly applied to femoral arteries immediately after injury as described in our previous studies.^48–50^ Similar to our previous observation,^48^ perivascular delivery of lentiviral particle and microRNA LNA inhibitor using 30% pluronic gel resulted in a specific local transfection, particularly in media VSMC layer in injured artery, as evidenced by GFP immunostaining (see Supplementary material online, *Figure S12A*) and RT-qPCR (see Supplementary material online, *Figure S12B*) analysis, respectively. Both snhg18 and miR-22-3p expressions in other tissues including heart, lung, and liver were not affected by perivascular delivery of lenti-snhg18 and miR-22-3p LNA inhibitor (see Supplementary material online, *Figure S12C*). Moreover, compared to controls, locally enforced expression of snhg18 in the injured arteries significantly increased snhg18, miR-22-3p, and VSMC contractile genes (Acta2 and Myh11), but dramatically decreased miR-22-3p target genes (Mecp2, Evi1, and Hdac4) and the cell proliferation marker gene Pcna (*Figure 6A*). Importantly, the modulatory effects of snhg18 overexpression on these genes were blunted by miR-22-3p inhibition (*Figure 6A*). Consequently, whilst vascular injury produced a thick neointima after 28 days in the mice treated with control gene/miRNA vectors, local ectopic expression of snhg18 significantly reduced neointima formation, as evidenced by a decrease in the intimal area and intima/media ratio in injured arteries treated with the snhg18 overexpression lentivirus (*Figure 6B and C*). As expected, the inhibitory effect of snhg18 on injury-induced neointimal hyperplasia was abolished by miR-22-3p inhibition (*Figure 6B and C*), confirming that modulation of miR-22-3p is responsible for the beneficial effect of snhg18 on injury-induced neointima formation. To further examine if there is an additive effect between snhg18 and miR-22-3p overexpression on injury-induced neointima formation, injured femoral arteries were embedded with 100 μL of 30% pluronic gel containing lentivirus (Lenti-GFP or snhg18) and miRNA AgomiRs (Cel-miR-67 or miR-22-3p), respectively. As previously documented,^48^ we observed a protective effect with miR-22-3p overexpression alone on injury-induced neointima formation, and such a protective effect was slightly enhanced by co-overexpressing snhg18 and miR-22-3p in injured arteries (*Figure 6D–F*), reinforcing the notion that up-regulated miR-22-3p expression by snhg18 is the main mechanism underlying the beneficial effect of snhg18 overexpression on injury-induced neointima formation.

### 3.8 Functional implication of SNHG18 in human VSMCs and arterial remodelling

**Figure 7:**
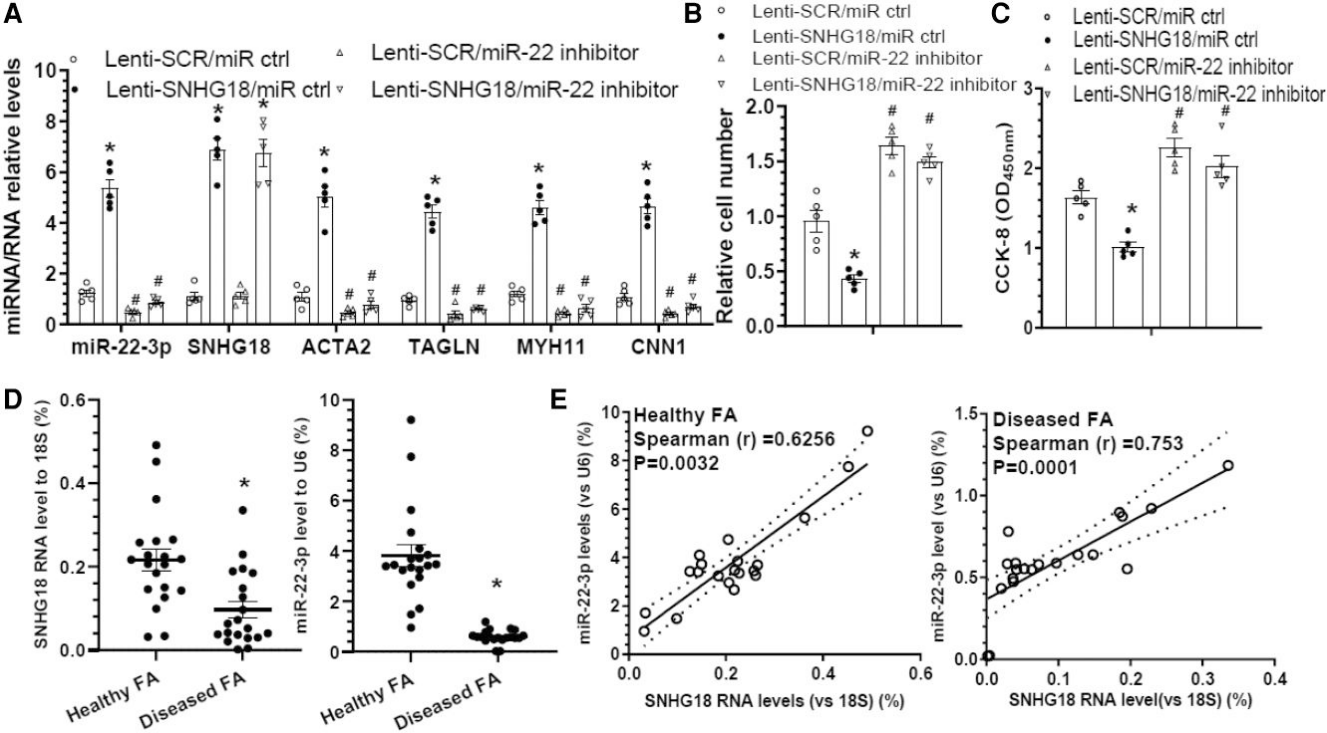
Functional implication of snhg18 in human VSMCs and arterial remodelling. Human VSMCs infected with control (Lenti-SCR) or human SNHG18 overexpression (Lenti-SNHG18) lentivirus were transfected with control (miR ctrl) or miR-22-3p inhibitor as indicated, followed by serum starvation for 24 h. After this, the cells were subjected to RT-qPCR analysis (*A*), cell count (*B*), and CCK-8 assay, respectively. Data and error bars represent mean ± SEM of five (*n* = 5) independent experiments. *P < 0.05 (vs. Lenti-SCR), ^#^*P* < 0.05 (vs. miR ctrl) (two-way ANOVA with a post-hoc test of Tukey’s analysis in A–C). (*D–E*) Expression profiles of SNHG18 and miR-22-3p in the healthy and diseased human arteries. Healthy FA specimens (*n* = 20) from patients without peripheral arterial diseases and diseased FA specimens (*n* = 20) from patients with peripheral arterial diseases were collected and subjected to RT-qPCR analyses. Expression levels of SNHG18 gene (relative to 18 s, %) and miR-22-3p (relative to U6 RNA, %) were presented in D. *P < 0.05 (vs. healthy FA, unpaired *t*-test). (*E*) Spearman rank correlation analyses were performed to characterize the relationships between the gene expression levels of SNHG18 and miR-22-3p in healthy FA and diseased FA specimens, respectively.

To translate our key findings from mice into humans, the potential role of the SNHG18/miR-22-3p signalling axis in human VSMC function was examined. Specifically, human VSMCs were co-transfected with a human SNHG18 overexpression vector and/or a miR-22-3p inhibitor, and subjected to serum starvation for 24 h, followed by various analysis. RT-qPCR data showed that while VSMC contractile marker genes were significantly increased and decreased by SNHG18 overexpression and miR-22-3p inhibition, respectively, the promotive effects of SNHG18 on VSMC marker gene expressions were abolished by miR-22-3p inhibition (*Figure 7A*). Moreover, while SNHG18 overexpression and miR-22-3p inhibition alone significantly decreased and increased human VSMC proliferation, respectively, such modulatory effects disappeared in the presence of a miR-22-3p inhibitor (*Figure 7B and C*). These data confirm that SNHG18 regulates human VSMC functions through upregulation of miR-22-3p.

To further support the translational value of these findings, RNA samples of human femoral arteries collected in our previous study^48^ were used to explore a potential role for the SNHG18/miR-22-3p signalling axis in human atherosclerotic intimal hyperplasia. RT-qPCR data showed that compared with healthy arteries, decreased gene expression levels of SNHG18 and miR-22-3p were observed in the diseased femoral arteries (*Figure 7D*). Moreover, we observed a significant positive relationship between SNHG18 and miR-22-3p in both healthy and diseased femoral arterial specimens (*Figure 7E*). Altogether, these data provide critical information about the functional relevance of the SNHG18/miR-22-3p signalling axis in human arterial remodelling.

## 4. Discussion

VSMC phenotypic modulation is not only essential for embryonic vascular formation and development, postnatal vascular growth, vascular homeostasis, and regeneration, but also critical for driving pathological arterial remodelling in response to injury and various external pathogenic stimuli. Although the underlying signalling pathways of VSMC phenotype switching have been extensively explored, our understanding of VSMC phenotypic modulation and its contribution to pathological arterial remodelling is still far from complete. Through our combined efforts we have been able to delineate a novel underlying mechanism for VSMC contractile-synthetic phenotype switching. Specifically, we show that SNHG18 expression is tightly modulated in both murine and human injured or diseased arteries, and demonstrate that SNHG18 promotes a VSMC contractile phenotype by up-regulating miR-22-3p. We also confirm that SNHG18 promotes miR-22 biogenesis and miR-22-3p generation by modulating the interactions between ADAR2, DGCR8, and Pri-miR-22. Importantly, we elucidate a potential therapeutic avenue for treating post-angioplasty restenosis by manipulating the SNHG18/miR-22-3p signalling axis. From a translational perspective, we also provide compelling evidence to support a potential role for the SNHG18/miR-22-3p signalling axis in human arterial remodelling.

Phenotypic modulation of VSMCs from a differentiated, ‘contractile’ state to a dedifferentiated, ‘synthetic’ phenotype, is associated with a decline in VSMC contractile gene expression and an increase in cellular proliferation and migration.^56^ It is one of the principal mechanisms of pathological arterial remodelling and post-angioplasty restenosis in mice and humans.^55,56^ A similar phenomenon can be observed in cultured VSMCs under conventional culture conditions^71,72^ with or without PDGF-BB treatment. Interestingly such phenotypic transition can be reversed through serum depletion/starvation,^59^ as well as with TGFβ1 treatment.^60,73,74^ By using various well-established *in vivo*, *ex vivo*, *in vitro* models of VSMC phenotypic modulation, we have shown that SNHG18 expression is closely modulated during VSMC contractile-synthetic phenotype switching. However, the molecular mechanisms regulating snhg18 expression in VSMCs are sparse. SNHG18 has been originally documented to be expressed in multiple cancers, with divergent roles in different cancers.^31–36^ In this study, we have shown that snhg18 can also be induced and transcriptionally regulated by TGFβ1 in VSMCs. It has been previously reported that SNHG18 is transactivated by megakaryocytic leukaemia 1 in non-small cell lung cancers^33^ and E2F transcription factor 1 in glioma.^75^ However, our unpublished data showed that none of these were impacted in serum-starved VSMCs by TGFβ1 treatment. Instead, we confirmed a critical role for Sp1 in SNHG18 transcriptional regulation in VSMCs. In consistent with our findings, we have previously documented an important role for Sp1 in regulating VSMC contractile gene expression.^76^ Moreover, it has been reported that Sp1 is lowly expressed in the model rats with carotid balloon injury, and Sp1 overexpression reduces injury-induced neointimal formation and restenosis rate.^77^ Therefore, findings from the study and previous studies clearly infer a functional role for TGFβ1/Sp1/SNHG18 signal axis in controlling and sustaining VSMC contractile phenotype as well as in preventing injury-induced adverse arterial remodelling.

Apart from Sp1, we also provide evidence to support a regulatory role for transcription factor SMAD3/4 in TGFβ1-mediated SNHG18 upregulation. Similar to Sp1, SMAD3 also plays a critical role in regulating VSMC-specific marker genes expression and VSMC differentiation from cardiovascular progenitor cells.^78^ Importantly, it has been recently reported that VSMC-specific SMAD3 controls VSMC phenotypic modulation in atherosclerotic plaque.^79^ These findings suggest a possible role for TGFβ1/SMAD3/SNHG18 signal axis in VSMC phenotypic modulation in the context of atherogenesis, which warrants further investigation.

The information about the cellular functions and potential implications in human diseases for SNHG18 in the literature is scant, with only handful of cancer studies reporting a diverse role for SNHG18 in various cancers.^31–36^ Specifically, SNHG18 was found to be up-regulated in glioma cells and promote radioresistance^31^ and motility^32^ of glioma cells. Another study showed that SNHG18 accelerates glioma progression by regulating the miR-338-5p/FOXD1 (forkhead box D1) axis.^75^ Similarly, it has been reported that SNHG18 facilitates non-small cell lung cancer growth and metastasis by modulating the miR-211-5p/BRD4 (bromodomain-containing protein 4) axis.^33^ Conversely, SNHG18 has been suggested as a tumour suppressor and potential diagnostic biomarker in hepatocellular carcinoma.^35,36^ To our knowledge, the current study is the first to document a functional role for SNHG18 in VSMC contractile-synthetic phenotype switching in the context of vascular diseases. We observe a decrease level of SNHG18 in diseased human arteries and a positive association between SNHG18 and miR-22-3p in both healthy and diseased human femoral arteries, and provide first-hand evidence to support the therapeutic potential of SNHG18/miR-22-3p signal axis in preventing injury-induced neointima formation.

Interestingly, we found that miR-22-3p expression was apparently regulated by SNHG18 in the context of VSMC contractile-synthetic phenotype switching. More importantly, by performing multiple biochemical and functional studies we confirm that miR-22-3p induction is essential for SNHG18-mediated VSMC contractile-synthetic phenotype switching. It is worth noting that the expression level of another miRNA, miR-34a-5p, was slightly but significantly upregulated by SNHG18 overexpression. Therefore, it would be interesting for us to further examine whether or not this miRNA is another target of SNHG18 in the context of VSMC phenotype switching, and if so, how this miRNA is regulated by SNHG18 in our future study.

However, how does SNHG18 regulate miR-22-3p in VSMCs? Functioning as a competing endogenous RNA to regulate other RNA transcripts including miRNAs is one of the key mechanisms through which lncRNAs regulate cellular functions and disease pathogenesis. Indeed, previous studies in cancers have shown that SNHG18 promotes FOXD1 expression by decoying miR-338-5p,^75^ suppresses nucleocytoplasmic transport of α-enolase,^32^ and inhibits semaphorin 5A^31^ in glioma cells. Moreover, SNHG18 exerts its prometastatic effects on non-small cell lung cancer cells through repression of miR-211-5p and induction of BRD4.^33^ If a similar mechanism underpinning miR-22-3p regulation by SNHG18 existed in VSMCs, we would have expected a down-regulation of miR-22-3p in SNHG18-overexpressing VSMCs since SNHG18 could decoy or suppress miR-22-3p. Our observation that miR-22-3p is dramatically up-regulated by SNHG18 overexpression in VSMCs refutes such a mechanism in VSMCs. Meanwhile, increasing miR-22-3p by SNHG18 in VSMCs also suggests that SNHG18 up-regulates miR-22-3p expression at a transcriptional level or during miRNA bioprocessing from a primary and/or precursor to mature transcript. Indeed, our data showed that the expression level of Pre-miR-22 not Pri-miR-22 was increased by SNHG18 overexpression, indicating that SNHG18 regulates miR-22 not through a transcriptional mechanism, but by facilitating the bioprocessing step between Pri-miR-22 and Pre-miR-22. This hypothesis was further strengthened by other observations that SNHG18 is mainly enriched in the nuclei of VSMCs, and no direct interaction was observed between SNHG18 and Pre-miR-22. We, therefore, speculated there is one or more RNA binding proteins functioning as the signalling intermediary between SNHG18 and Pri-/Pre-miR-22, or serving as a docking site for both RNA transcripts. By using the bioinformatics tool, together with multiple functional studies including RNA pull-down, RIP, RNA-EMSA, and gene mutations, we provide comprehensive evidence to show that ADAR2 prevents or interferes the binding of DGCR8 to the Pri-miR-22 transcript, which in turn decreases the miRNA bioprocessing efficiency of the ribonuclease III enzyme Drosha, thereby inhibiting Pre-miR-22 bioprocessing and miR-22-3p generation.

It is well-known that ADAR2 can deaminate adenosines to inosines (also referred to as A-to-I RNA editing) in double-stranded RNAs including primary/precursor miRNAs to recode the open reading frames or cause conformational changes in edited RNA transcript, thereby modulating its target gene expression and function.^80,81^ Accordingly, it is plausible to assume that ADAR2-mediated A-to-I RNA editing in coding and non-coding RNAs may affect many basic biological processes including introducing amino acid mutations into coding region, affecting the RNA structure to alter RNA biological processing, stability, and biosynthesis, causing miRNA retargeting as well as mRNA alternative splicing.^82^ A critical regulatory role for ADAR2 in miRNA biogenesis and regulation has been drawn from Adarb1 (for ADAR2) and Adar (for ADAR1) gene knockout mice. Specifically, Vesely *et al*.^66,67^ conducted RNA sequencing analysis to compare the relative frequencies and sequence composition of miRNAs in Adarb1^−/−^ and/or Adar^−/−^ mice to wild-type mice, and they found that Adarb1 deficiency leads to a reproducible change in abundance of a large number of miRNAs. The expression of these miRNAs is primarily dependent on Adarb1 since additional deletion of Adar produces little impact on the mature miRNA repertoire. It has also been reported that ADAR2 mainly inhibits miRNA generation since Adarb1 deficiency leads mostly to an upregulation of mature miRNA sequences. Moreover, it has been shown or suggested that A-to-I RNA editing events appear to be unrelated to the changes in miRNA abundance for majority of the deregulated miRNAs observed in Adarb1^−/−^ mice.^66–68^ Consistent with these findings, we also observed a very low level of A-to-I editing within the Pre-miR-22 transcript, and found that ADAR2-mediated A-to-I editing plays an insignificant role in miR-22 bioprocessing and miR-22-3p production, suggesting that miR-22-3p maturation is negatively regulated by ADAR2 in an editing activity-independent manner.

In summary, we have collectively demonstrated that SNHG18 is a novel regulator of VSMC contractile-synthetic phenotype switching and vascular neointimal lesion formation, and that SNHG18 controls VSMC contractile-synthetic phenotypic modulation and inhibits injury-induced arterial remodelling via its target gene miR-22. Therefore, our findings offer a novel therapeutic opportunity for treating vascular diseases by targeting the SNHG18/miR-22-3p signalling axis. Moreover, we have also provided comprehensive evidence to show that miR-22-3p generation is finely tuned process regulated by SNHG18/ADAR2/DGCR8 in VSMCs. In VSMCs with a dedifferentiated/‘synthetic’ phenotype, SNHG18 is low or down-regulated by PDGF-BB or other pro-atherogenic stimuli, which allows ADAR2 to compete with DGCR8 in binding to Pri-miR-22 transcript and even displace DGCR8 from its interaction with Pri-miR-22, thereby inhibiting or preventing miR-22 biogenesis. In contrast, in VSMCs with a differentiated/‘contractile’ state, SNHG18 is high or up-regulated by TGFβ1. Up-regulated SNHG18 sequesters ADAR2 from ADAR2/DGCR8/Pri-miR-22 complex, allowing miR-22 to undergo full bioprocessing steps with high efficiency, eventually increasing mature miR-22-3p production. Subsequently, increased miR-22-3p promotes a contractile VSMC phenotype via its target genes, MECP2, HDAC4, and EVI1.

**Translational perspective**

In this study, we confirm that lncRNA SNHG18 plays a critical role in governing VSMC contractile phenotype and preventing injury-induced neointimal hyperplasia. Since VSMC phenotypic modulation and dysfunctions are the fundamental causes for many cardiovascular pathological conditions including post-angioplasty restenosis, atherosclerosis, in-stent restenosis, peripheral arterial diseases, hypertension, and stroke, local and systemic modulation of this newly identified signal axis (SNHG18/miR-22-3p) could represent as a novel therapy for these diseases.

## Supplementary Material

cvae055_Supplementary_Data

## Data Availability

Some data may not be made available because of privacy or ethical restrictions. All remaining data are contained within the article, and the data that support the findings of this study are available from the corresponding author upon reasonable request.
