# Supplementary material for: Small nucleolar RNA host gene 18 controls vascular smooth muscle cell contractile phenotype and neointimal hyperplasia
Source: Cardiovasc Res. 2024 Mar 18;120(7):796–810. doi: 10.1093/cvr/cvae055 (PMC11135647; doi:10.1093/cvr/cvae055)

Full WB images

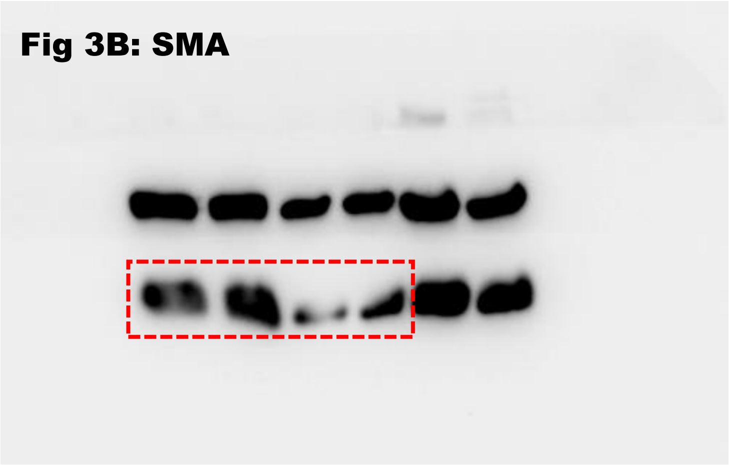

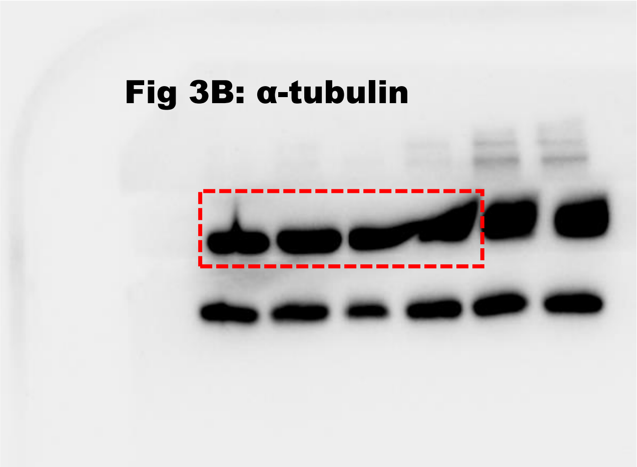

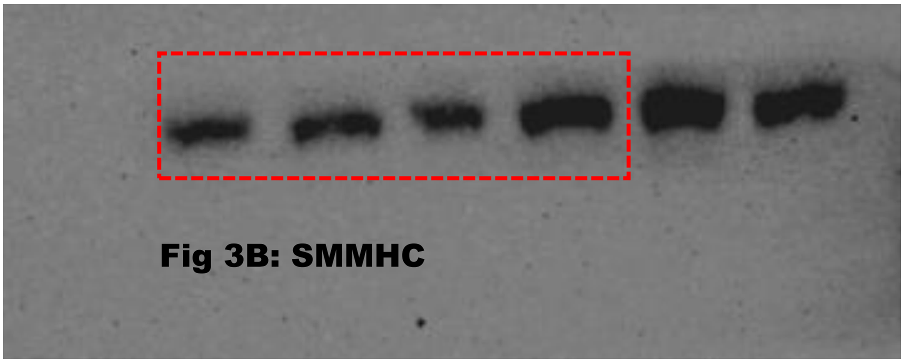

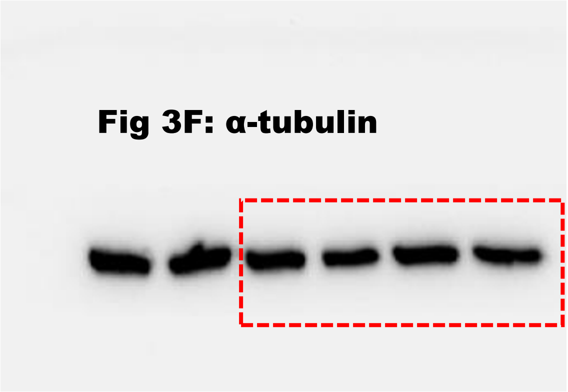

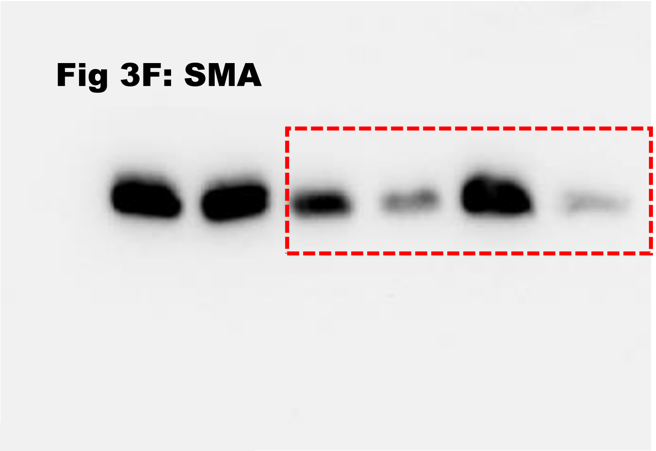

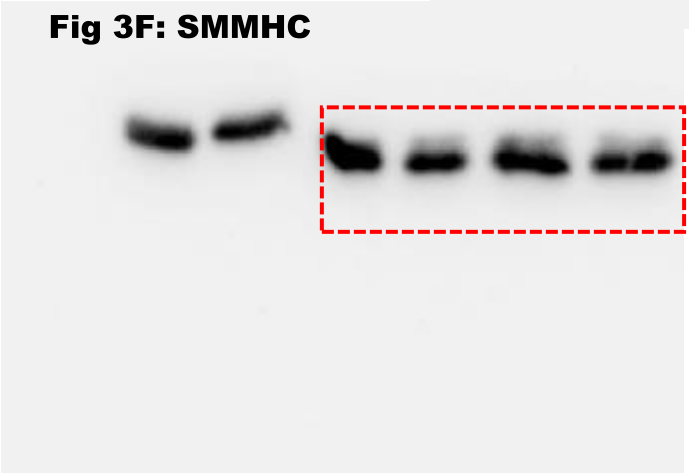

Fig 4D: SMA

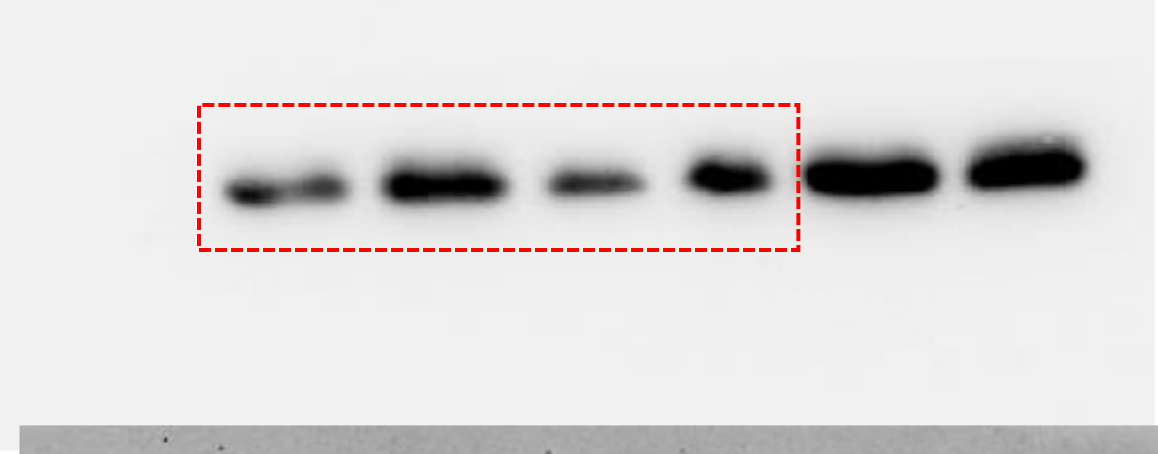

Fig 4D: SMMHC

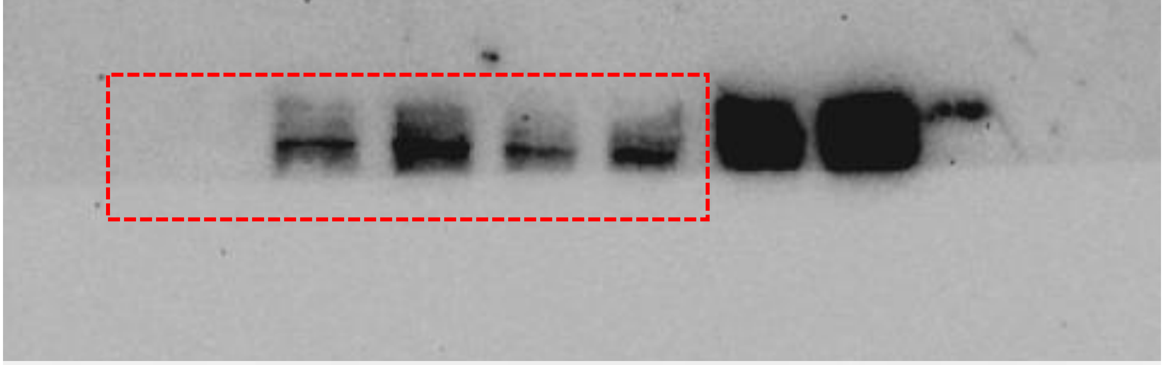

Fig 4D: α-tubulin

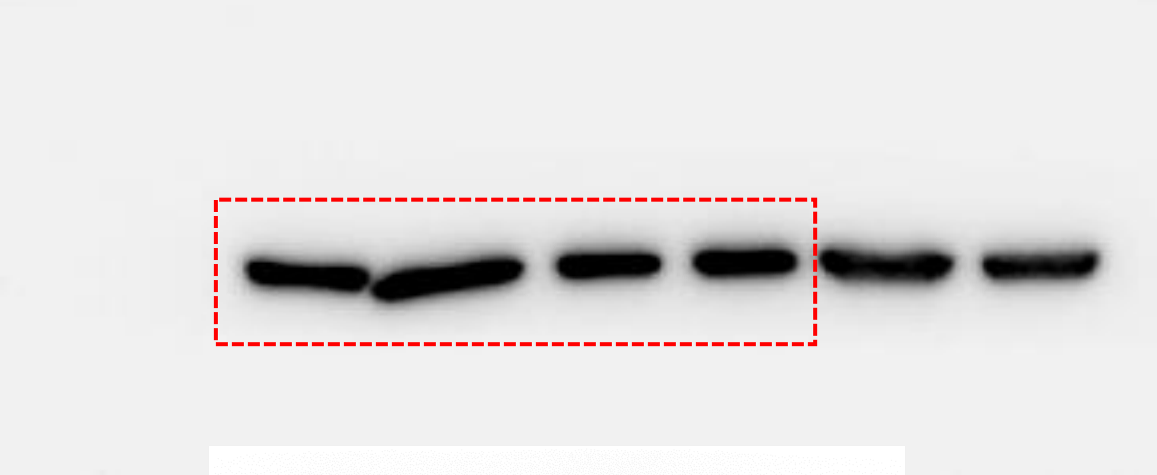

Fig 5I: RNA-EMSA

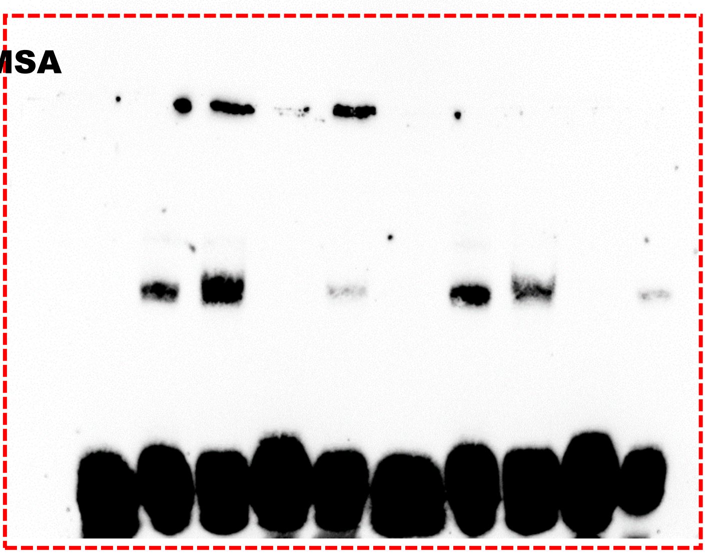

Supplement: cvae055_Supplementary_Data [file cvae055_supplementary_data.zip › Full WB images for CVR-2023-1492 (2).pdf]
