# Supplementary material for: Small nucleolar RNA host gene 18 controls vascular smooth muscle cell contractile phenotype and neointimal hyperplasia
Source: Cardiovasc Res. 2024 Mar 18;120(7):796–810. doi: 10.1093/cvr/cvae055 (PMC11135647; doi:10.1093/cvr/cvae055)
Supplement: cvae055_Supplementary_Data [file cvae055_supplementary_data.zip › Snhg18 in NI_SI_DeNovo.doc]

***SUPPLEMENTARY DATA***

**SNHG18 controls vascular smooth muscle cell contractile phenotype and neointimal hyperplasia**

Kaiyuan Niu^1#^, Chengxin Zhang^2#^, Mei Yang^1,3^, Eithne Margaret Maguire^1^, Zhenning Shi^1^, Shasha Sun^3^, Jianping Wu^1^, Chenxin Liu^1^, Weiwei An^1^, Xinxin Wang^2^, Shan Gao^4*^, Shenglin Ge^2*^ and Qingzhong Xiao^1,2,4*^

(**Running Title:** Niu, et al. SNHG18 in VSMC functions and neointimal hyperplasia)

**Methods & Materials**

**Materials**

Antibodies against alpha smooth muscle Actin (SMA) (rabbit IgG, ab5694; mouse IgG, ab7817), smooth muscle Myosin heavy chain 11(SMMHC) (rabbit IgG, ab124679), GFP (rabbit IgG, ab290), and DGCR8 (rabbit IgG, ab191875) were purchased from Abcam, UK. Antibodies against SMA (mouse IgG, A5228) and α-Tubulin (mouse IgG, T6074) were from Merck, UK. Antibody against Sp1 (rabbit IgG, Catalog # MA5-35331) was from ThermoFisher Scientific UK. Antibody against ADAR2 (Mouse IgG, sc-73409) was from Insight Biotechnology Ltd UK. All secondary antibodies and other materials were from ThermoFisher Scientific UK unless specifically indicated.

**Cell isolation, culture and treatments**

Primary murine VSMCs were isolated from aorta (ascending to abdominal aorta) of eight-week old C57BL/6 mice with both sexes, and routinely maintained in DMEM supplemented with 10% FBS as described in our previous studies^1-8^. Human aortic smooth muscle cells were purchased from PromoCell GmbH (C-12533) and cultured in VSMC growth medium 2 (PromoCell GmbH, C-22062) according to the manufacturer's instructions. Both murine and human VSMCs between P3 and P8 were treated with various atherogenic stimuli as described in the previous studies^1-8^. Briefly, VSMCs were serum starved for 24 hours (0% FBS), followed by an incubation with vehicle control, PDGF-BB (10 ng/ml), or TGFβ1 (5 ng/ml) as indicated for additional 24 hours.

**Collagen-based** **Cell Contraction Assay**

VSMC contraction assays were performed using Collagen-based Cell Contraction Assay kit (antibodies-online, Catalog No. ABIN2344900) per manufacturers’ instructions. Briefly, primary murine VSMCs with indicated treatments were harvested and re-suspend in DMEM at 2.5 x 10^6^ cells/mL and mixed with cold collagen gel working solution. Half mL of the cell-collagen mixture was added into each well in a 24-well plate and incubated 1 hour at 37 °C. After collagen polymerization, 1.0 mL of culture medium was added atop each collagen gel lattice. After cultured for two days, contraction was initiated by gently releasing collagen gels from the sides of the culture dishes with a sterile spatula. Cell contraction index was measured and quantified as the percentage (%) of volume change between before and after collagen gel release (Vol_before_–Vol_after_)/Vol_before_ x 100).

**Fluorescence *in situ* hybridisation (FISH)**

Similar to our previous study^5^, FISH was conducted to detect the cellular location of snhg18 in VSMCs using miRCURY LNA miR ISH Optimization Kit (FFPE) and TSA Plus Fluorescence Kit per manufacturers’ instructions. Briefly, VSMCs were fixed in 4% paraformaldehyde/PBS for 10 minutes, and permeabilized with 0.05% triton-X100/PBS for 15 minutes at room temperature (RT), respectively. After washing in PBS for 5 minutes, cells were incubated with 3% H_2_O_2_ for 15min, washed in PBS (3 min x 3), and treated with Proteinase-K (4ug/L) at 37°C for 1 minute. After washing, the cells were incubated with 50 μL hybridization solution containing 40 nM (3.2 μl) double-fluorescein LNA™ scramble (5’-FAM/GTGTAACACGTCTATACGCCCA/3’-FAM) or snhg18 gene specific (5’-FAM/ATGCTACAGAGACATCAAACTT/3’-FAM) probe (both are purchased from Qiagen) at 60°C for 60 minutes. After which, cells were washed in sodium citrate dehydrate (SSC) solution at 60°C (5xSSC, 5 minutes, once; 1xSSC, 5minutes, twice; 0.2xSSC, 5 minutes, twice), and finally in 0.2xSSC buffer at RT for 5 minutes. Thereafter, cells were blocked with blocking solution (5%BSA) for 15 minutes, followed by incubation with HRP-conjugated anti-FAM antibody (1:100 in 1% BSA blocking solution) at 4°C overnight. After washing in PBST (3 minutes x 3), cells were incubated with TSA^TM^ Fluorescein System (Perkin Elmer, NEL741001KT) at RT for 10 minutes, and then mounted with SlowFade® Gold antifade reagent with DAPI (Life Technologies, S36938).

**Aortic tissues immunofluorescence (IF) and FISH analysis**

Arteries were fixed, sectioned, and subjected to routine IF staining with the primary antibodies (SMA, GFP, Sp1 or corresponding IgG control), followed by incubation with an appropriate Alexa Fluor-conjugated secondary antibodies. Images were taken using a laser scanning confocal microscope (Zeiss LSM 510 Mark 4) and Zen 2009 image software. The fluorescence intensity for target proteins and DAPI was quantified using the Image J software. In some cases, FISH assays were simultaneously conducted using miRCURY LNA miR ISH Optimization Kit (FFPE) and TSA Plus Fluorescence Kit as described above to detect the spatial location of snhg18 in murine arteries. Briefly, aortic sections were washed thrice with PBS for 3 minutes and permeabilized with 100 μL of 0.1% triton-X100/PBS for 15 minutes. After washing twice with 100 μL PBS for 5 minutes each, sections were incubated with 3% H2O2 for 15min, washed in PBS (3 min x 3), and treated with Proteinase-K (4 ug/L) at 37°C for 5 minutes. After washing, aortic sections were incubated with 50 μL hybridization solution containing denatured probe mixture at 60°C for 4 hours, followed by similar steps as described above for remaining procedures.

**Real time quantitative PCR (RT-qPCR) for mRNA and miRNAs**

RT-qPCR analysis for mRNA and miRNA detection was conducted as described previously. Briefly, total RNA containing small RNAs was extracted from murine aortas, human femoral arteries or cells using Trizol reagent (Sigma) according to the manufacturer's instructions, and subjected to DNase I (Sigma) digestion to remove potential DNA contamination. Reverse transcription for mRNAs and miRNAs was performed using an Improm-II^TM^ RT kit (Promega, Madison, WI, USA) and the NCode™ VILO™ miRNA cDNA Synthesis Kit (Invitrogen, A11193-051), respectively, per manufacturer's instructions. The resultant cDNA was diluted to a working concentration of 5ng/μl and stored at -20ºC. NCode™ EXPRESS SYBR^®^ GreenER™ qPCR SuperMix Universal was used in miRNA RT-qPCR. Relative mRNA or miRNA expression level was defined as the ratio of target gene expression level or miRNA expression level to 18S or U6 snRNA expression level, respectively, with that of the control sample set as 1.0. Primers were designed using Primer3-BLAST (National Center for Biotechnology Information, USA) and the sequence for each primer was listed in **supplementary Table S1**.

**Isolation of nuclear and cytoplasmic RNAs**

VSMCs were subjected to serum starvation for 24 hours, and one-third of cells were used for total RNA isolation. The remaining two-third cells were used for isolation of nuclear and cytoplasmic RNAs using the SurePrep™ Nuclear or Cytoplasmic RNA Purification Kit Product (Cat. # BP2805-25) per manufacturer's instructions. The resultant RNAs were subjected to RT-qPCR analysis with primers listed in **supplementary Table S1**. GAPDH and U6 snRNA were used as control for cytoplasmic and nuclear RNAs, respectively. Data were presented as the relative RNA levels in nuclear or cytoplasmic fractions over their respective level in total RNAs.

**miRNA inhibitor or antisense locked nucleotide acid (LNA) GapmeRs transfection**

Either miR-22-3p inhibitor, miRNA control inhibitor, control scramble LNA or SNHG18 specific LNA (25nM, final concentration) were transfected into VSMCs using Turboffect Transfection Reagent (ThermoFisher Scientific UK) according to the manufacturer's instructions. With Cy3™ Dye-Labeled Pre-miR Negative Control #1 (AM17120, Thermo Fisher Scientific Inc. UK), we detected over 70% transfection efficiency for miRNA transfection in VSMCs previously reported^6^. MiR-22-3p and negative controls were purchased from ThermoFisher Scientific UK, and both control scramble LNA and SHNG18 LNA GapmeRs from Qiagen, Denmark.

**VSMC proliferation and migration assays**

VSMC treatment and functional analysis were similar to our previous studies^1-8^. Specifically, for manually cell counting, VSMCs were plated (5x10^4^ per well) and cultured in 12 well-plate. Once reached 80-90% confluence, cells were subjected to various treatment as indicated in the respective figure legend. Thereafter, VSMCs were detached and manually counted using a hematocytometer. The relative cell number was defined as the ratio of treatments to controls, with that of the control sample set as 1.0. For the CCK-8 assay, VSMCs with various gene manipulations as indicated in figure legend were plated (0.75 x10^4^ per well) and cultured in 96 well plates overnight, followed by serum starvation for 24 hours, with or without additional treatment. After then, cell viability or proliferation was evaluated using Cell Counting Kit-8 (CCK-8) kit (Sigma/Merck, 96992-500TESTS-F) according to the manufacturer's instructions as described previously. The absorbance of the samples representing cell proliferation/viability was measured by a microplate reader at 450nm (OD_450_). For the trans-well migration assay, VSMCs with various gene manipulations as indicated in figure legend were subjected to serum starvation for 24 hours. Serum-starved cells were harvested by trypsinization. An aliquot (250,000 cells/200µl) of the cells in serum-free DMEM was dispensed into the trans-well inserts (8µm pore size, Greiner Bio-One Ltd, UK. Item number: 662638) pre-coated with 0.5% gelatin (Sigma, G1393), and serum-free DMEM containing 30ng/ml PDGF-BB was placed in the lower chamber. The trans-well plates were incubated at 37°C in a 5% CO2 incubator for 12~18 hours. Non-migrated cells in the top insert were carefully removed by cotton swab, and the migrated cells in the bottom side were stained with Crystal Violet dye. Images were captured at five fixed locations (right, bottom, left, up and centre), and migrated cells were counted by two experienced investigators blinded to the treatments.

**Immunoblotting**

Cells were harvested and lysed in lysis buffer (50mM Tris-Cl pH 7.5, 150mM NaCl, 1 mM EDTA pH 8.0) supplemented with protease inhibitors and 0.5% Triton and sonicated to obtain whole cell lysate. 40 μg of protein was separated by SDS-PAGE with 4%~20% Tris-Glycine gel (Invitrogen, Carlsbad, CA, USA) and subjected to standard Western blot analysis. The blots were subjected to densitometric analysis with Image J software. Relative protein expression level was defined as the ratio of target protein expression level to α-tubulin expression level with that of the control sample set as 1.0.

**Generation of snhg18 gene promoter and Sp1 binding site mutants**

Mouse snhg18 gene promoter DNA fragments containing either wild type, or mutated Sp1 binding sites were synthesized by GenScript (Nanjing, China) and sub-cloned into the NheI and HindIII sites of the pGL4-luciferase-reporter-vector (Promega, UK), designated as pGL4-snhg18-WT, and pGL4-snhg18-Mut(1-4), respectively. Similarly, DNA fragments without the corresponding SMAD3/4 binding site(s) were also synthesized by GenScript (Nanjing, China) and sub-cloned into the NheI and HindIII sites of the pGL4-luciferase-reporter-vector (Promega, UK), designated as pGL4-snhg18-d1, -d2 and d1/2, respectively. All vectors were verified by DNA sequencing.

Below is the DNA sequence for wild type mouse snhg18 gene promoter (NC_000081.7:c32246808-32240714 chromosome 15, GRCm39), with the underlined DNA sequences predicted as the binding sites 1-3 for transcription factor Sp1 (Sp (1-3). Sequences with red font were predicated as the binding sites for SMAD3/4.

TCTCCGGGACGCCGCAGGCGCAGGAAAGTTAAAACCCAACTCAAGGCAACCGCATCCAGGTACAAACAAC

AAAAAAGGACGTGAAAGCGCTTCCCGCCAAAGCCCGGGGCGGGAGGTGAGGGGTACCGGGACCGGGACCT

ACCCGCTCTGGGCCCAATTCCAAGGGCGATCGCCGGAGATCACTCCCCTCCAACAGTTCCTCGGAATATC

CTGAGCACCGATCTTCTCCTGGCCCCAGACCGTGAACCCGGGTATGAAATGCGTGCGGCTGGGCTGCCCG

CAGACGGGGGCTCTGCGATTGGAGCTCATCCTGCTGAAGCCCCAGGCTTCGCCATCCCTGCCTTAGTCTT

CCTCAGCAAAACACCCTGGCGGAGTTGTGCCCTGGTCCCACTAACCCCAGATTTTCACGAACACCGCAGA

GCCCCTGTGCAGAGAGCAGGAAAATAAATGTCCCCTAGTCCACCGTGCAGGGAGGTAGGCGATTCCGACC

ACCAATCCCGGTTCCAGATCCCACCAAGCAAGCCTGGCCTAGCGCGGATTACGCACACAGATCTCCACGG

GTACCTGAAAGGTGCTGGCCGAGGTGCAGGCTTGCCCACTGACCGGTCGCACACCCGGACCTTCTCAATC

CACGCTCGTATCCAGGGAAAGGCTAAGACACAAACTTCCAGCCCGTGCACTATCTCCACTGCGACCGCCC

CCGCTTTGATGGTAGTCCTGCACGCGCCTCTGCCAGGTTGTCCCCACCCAGGTGTCTGCAGAGTGACCCT

ACCAGAGACTTACACCACCCGCAAATGGACCAGTGCCGAGTCCAGTTCACACGTGCAGGGTCTCAGCTAT

GACACTCAGGGACTCTGCGGGGAGAGAGCTCACCAGATGCCGGGCGCGTTCCTCCTGCTAGTCATCCAGC

GCCCCGGGGCCCGGAGCTGCTCCAAGGGTCCTCTTCAGTGTACTACGAGGGAAGTCGAGGCAGCCGCCAC

CACTGCACCCCGCCACTTCTCCAGAGCTATCCTCCTCTCGCAGCGCGCCAGGCTGGGGACCAGCTAGTCT

GCCGGAGTCTCCAGTTGTGCAGCTGAGTGGACAGGCTCGCGCCGAGAGAGGGCGACCGGTGAGGTGGCAA

AGTTGGGTACCCTCTGCTTAGTAGCTGCGCGTGGGAACCAAGAACACCCCCAAAAAGGATTGCACGTGCC

GGGACGCCCCCGCAGTGGGAAGGTGGGATACAGAGGTCGCCACAAGGCTGGGGCCACCGTCAGCCAAGGG

CTGAGGAGCCCACCCGCGGCGGGAGCGCAGCGCGCGCGGGAGTGGGCACAGCGTGAGCAGCCGCTGCTGA

CGCCGCCTTCCTAGCGAGTAGACCAGCGCTGGGTGAGCGGGCCCAGAGCTCGGGGCGGGGAGCACAGGAC

CGCCTCGCCTCGCCCCGCCCCCGCCCCGCCCCCTCCGCCGCACCTCCCCTACTCCGCCCCTCCCGCGTGC

AAACTGAGCGCCCCACCCACCCCTGACCTTCCCAGGCCCCTCCTCTAGCCCCGCCTCTTCCCGAGATGGG

**Transient transfection of gene promoter reporters and luciferase assay**

Luciferase assays for snhg18 gene reporters were conducted as previously study. Briefly, VSMCs were co-transfected with individual gene promoters (pGL4-snhg18-WT, pGL4-snhg18-Mut1, pGL4-snhg18-Mut2, pGL4-snhg18-Mut3, or pGL4-snhg18-Mut4) (0.15 μg/2.5 x 10^4^ cells) and p*Renilla* (15 ng/2.5 x 10^4^ cells) using Turboffect Transfection Reagent (ThermoFisher Scientific UK) per manufacturer’s instructions. Transfected cells were subjected to various treatments as indicated in the respective figure legend. Dual-Luciferase Reporter Assay System was used for detecting luciferase and *Renilla* activities according to the protocol provided in the system. Relative luciferase unit (RLU) was defined as the ratio of Luciferase versus *Renilla* activity with that of the control (set as 1.0).

**Generation of gene overexpression and knockdown lentivirus**

Scrambled DNA (shown below) and DNA fragments for mouse (NR_038186) or human (NR_045196) SNHG18, wild type (NM_130895.3) or mutated (E396A, GAG to GCG) mouse Adarb1 were synthesized by GenScript (Nanjing, China) and sub-cloned into the ECoRI and NotI sites of the pCDH-CMV-MCS-EF1-Puro cDNA Cloning and Expression Vector (CD510B-1, Cambridge Bioscience, UK), designated as Lenti-SCR, Lenti-snhg18, Lenti-SNHG18, Lenti-Adarb1, and Lenti-Adarb1 (E396A), respectively. All vectors were verified by DNA sequencing. Control (Lenti-SCR or Lenti-GFP) and gene-specific overexpression pseudoviral particles were generated using Lenti-SCR, Lenti-GFP, Lenti-snhg18, Lenti-SNHG18, Lenti-Adarb1, or Lenti-Adarb1 (E396A) and packaging plasmids (pCMV-dR8.2 and pCMV-VSV-G), respectively, as described in our previous studies.

Below is the DNA sequence for Lenti-SCR (scrambled DNA for both mouse and human SNHG18 overexpression lentivirus):

TTAGAAGAGAGACAGATAGCTTCTTACCGGTGCGCCACCGTAGGCAGTACGATCGCACGCCCCATGTGAACGATTGGTAAACCCAGTGTCCTGTGAGCGACAAAAGCTTAAATGGGAAATACGCGCCCATAACTTGGTGCGAATACGGGTCGTAGCAATGTTCGTCTGACTATGATCTACATATTACAGGCGGTACGTCTGCTTTGGTCAGCCTCTAATGGCTCGTAAGATAGTGCAGCCGCTGGTGATCACTCGATGACCTCGGCTCCCCATTGCAACTACGGGGATTCTTGGAGAGCCAGCTGCGTTCGCTAATGTGAGGACAGTGTAGTATTAGCAAACGATAAGTCCCGAACTGGTTGTGACCTAACGAAAAGTGAACTTCATAATACGTGCTGTCCCACGCACATGGTAGATTTGGACAAAATTGAATGGAGTCTGATCAACCTTCACACCGATCTAGAATCGAATGCAGTGATCTCCCAGGTGCGAATCAAAAATACTAGGTAACTAGAGGATTTGCGACGTTCTAAGCGTTGGTCCATGTGAATCGCCATCCAGGATCACGTCGCCCTGAAAAAAAGATATCAGCAACTCTCCTCCTCAGCAGTCTGGTCTATGGAAACTACAGGACTAACCTTCCTAGCAACCGGGGGCTGGGAATCTGTGACATGAGTCAAGATATTTGCTCGGTAATCTATGCTCTAGGCATCTAACTATTCCCACTGCCTTAAGGGGGCCTGCGTTTTCTGCCTGTCGATCCATAGGACTCGTGTCAACGCGCAGGCTTAGTTCGAGATGAAATGTCCGGGGCCAAAGACAACCAGCATCTCGCGTCTTGCCTAACCCCCCTACATGTTGTTATAGACAATCAGTGGAAACCCAGTGCCAGACGATGGAATGACCTTAAGTCAGGCAGGAAATTAAAGGGAACGTATATTCAACGCAATGAAGCTGGAGGATTGGCGTGGGAATCGTGCTTCTGTCTAAGCAAGAATGGGTATGAGGTGGCAACCGTCCCCCTAGCGTACAGGGTGCACTTTGTAACGATTTGGGAGTCCAGAGACTCGCTGTTTTCGAAATTTCCCTTTAAGCGCGGGTATTGAACCAGGCTTATGCCCAAGATCGTAGCAAGCAGACTCAAACAAAATATATTTTGCCCGCGTTACAGACGAAACTAGTTGGAGGTTATGGAGCATACTAACACGTGGACGGCCACTGGTGAGTTGCTACACCCCTGCGGCAACGTTGAAGCTCCTAAATTACTCTGGCTGGATCCAAGCCGTAACACCCGTCTCACTTCATAACCGTTTGCAATTCACGGCTTGATCTAGACTGGATTGCCATTCTCTCAAAGTATTATGCAGGCCGGCGTACGCGTCCCATATAAACCTGTCATAACTTACCTGAGACTACTTGGAAATGTGGCTAGATCTTTGCCCACGCACCTAATCGGTCCACGTTTGGTTTTTAGGACCCGATGATCTTCAAAACACTGCA

As described in our previous study^16^, Sp1 and Adarb1 shRNA lentiviral particles were produced using MISSION shRNA Sp1 (SHCLNG- NM_013672,	TRCN0000071606, MISSION® shRNA Bacterial Glycerol Stock, Sigma) or Adarb1 plasmids DNA (SHCLNG-NM_130895,	TRCN0000086464, MISSION® shRNA Bacterial Glycerol Stock, Sigma) according to protocol provided. The shRNA Non Targeting control vector (SHC002) was used as a negative control (Non-target).

Lentiviral infection to specifically overexpress or knockdown target genes in VSMCs was performed as described in our previous studies with some modifications^16-18^. Briefly, VSMCs were plated 24 hours prior to infection in 6 well-plates at 37°C. One transducing Unit (TU) per cell or 2-3x10^5^ TUs pert well of lentivirus were added with 10μg/ml hexadimethrine bromide (H9268; Sigma), and incubated for 24 hours before further treatment. If required, the media was replaced with complete media containing 4μg/ml puromycin (P9620, Sigma) to select stable transductants. Fresh media containing puromycin was added at 2-3 day intervals for 7~10 days. Stably infected cells were split and frozen for future experiments.

**Chromatin immunoprecipitation (ChIP) assays.** The ChIP assays were performed as previously described. Briefly, serum-starved VSMCs treated with vehicle control or 5 ng/ml TGFβ1 were fixed, harvested, and sonicated. The sheared samples were incubated with antibodies raised against Sp1 (rabbit IgG, Catalog # MA5-35331), or rabbit IgG controls (5µg/immunoprecipitation). After pull-down using protein-G Dynabeads, the immunoprecipitates were eluted from the beads using 50 μl elution buffer. Immunoprecipitaed DNA was extracted, purified, and then used to amplify target DNA sequences by RT-qPCR using specific primers (**Online Table S1**). Promoter DNA enrichment with specific antibody was calculated using percent input method with that of the IgG control set as 1.0. PCR amplification of snhg18 gene intron was included as additional control for specific snhg18 gene promoter DNA enrichment.

**Morphometric analysis and quantification of lesion formation**

The procedure used for sample preparation and lesion quantification was similar to that described previously. Briefly, EEL (external elastic membrane), IEL (internal elastic membrane), and lumen areas were automatically measured on H&E stained cross sectional femoral artery segments using a computerized image analysis system (pixel^2^, Axiovision software) by two experienced investigators blinded to the treatments. Thereafter, media area, neointimal area, and their ratios were calculated. Four to five sections were analyzed per vessel sample.

**RNA immunoprecipitation (RIP) assay**

The RIP assays were performed as described previously. Briefly, VSMCs infected with control or gene-specific shRNA lentivirus were subjected to serum starvation for 24 hours and treated with 1% (v/v) formaldehyde at room temperature for 10 min. After quenching with glycine at room temperature, VSMCs were harvested, lysed and sonicated in the lysis buffer containing RNase inhibitor. The sheared samples were incubated with 5µg antibody against ADAR2 (Mouse IgG, sc-73409), DGCR8 (rabbit IgG, ab191875), or equal amount of mouse/rabbit IgG, together with protein-G Dynabeads® saturated with single-strand salmon sperm DNA. After digestion with DNAse, the immunoprecipitates were eluted from the beads using 100 μl elution buffer, and immunoprecipitaed RNA was extracted, purified, and then used to amplify target RNA sequences by RT-qPCR using specific primers (**Table S1**). RNA enrichment with specific antibody was calculated using percent input method with that of the IgG control set as 1.0. The relative level of RNA enrichment was defined as the ratio of RNA enrichments in VSMCs infected with Lenti-snhg18 or sh-Adarb1 to VSMCs infected with Lenti-SCR or sh-NT, respectively, with later sample set as 1.0.

**Snhg18 RNA pull-down analysis**

RNA pull-down analysis was conducted as described previously^25^. Briefly, VSMCs with the indicated treatments were fixed in 1% paraformaldehyde solution in PBS for 10 minutes at RT, and quenched by adding 1/10 volume of glycine 1.25 M for 5 minutes at RT, respectively. After washing, cells were collected, lysed, and sonicated in the lysis buffer containing RNase inhibitor to generate RNA fragments with optimised length range between 200 and 800 bp. The sheared samples were incubated with 100 pmol of biotinylated oligonucleotide probes (scrambled probe: GTGTAACACGTCTATACGCCCA; snhg18 probe: ATGCTACAGAGACATCAAACTT) under moderate agitation on a tube rotator at RT for 5 hours, followed by an incubation with 50 µL of magnetic streptavidin beads supplemented with a RNAse inhibitor (200 U/mL) and a cocktail of proteases inhibitor (5 µL/mL) overnight at RT on a tube rotator. After washing, beads were digested with proteinase-K (20 mg/mL) for 45 minutes at 50 °C, 10 minutes at 95 °C, then on ice for 3 minutes. RNAs were eluted from beads and purified, followed by RT-qPCR analysis. RNA enrichment with specific probe was calculated using percent input method.

**Endonuclease V immunoprecipitation enrichment (EndoVIPER) and analysis of A-to-I edited RNAs**

EndoVIPER was performed as described previously. Briefly, 500 ng total RNAs extracted from VSMCs with the indicated treatment shown in the figure legend were incubated with 20 µl NEBNext® Magnesium RNA Fragmentation Module (New England Biolabs, cat. No. E6150S) for 1 minute. After then, the reactions were mixed with 2 μl of the 10× RNA Fragmentation Stop Solution on ice, and transferred into a 1.5 mL microcentrifuge tubes, which were sequentially added with 2.2 μL 3 M sodium acetate (pH 5.2), 2 μL 20 mg/mL glycogen, and 60 μL cold 100% ethanol. After mixing and incubation at −80 °C for an hour, samples were centrifuged (~17,000 x g) for 20 minutes in a refrigerated microcentrifuge, followed by washing with cold ethanol twice. Air-dried RNA pellets were reconstituted in 10 μl nuclease free water and incubated at 70 °C for 2 minutes to denature mRNA samples. Fragmented mRNA samples (~200–500 nt in length) were incubated with 40% glyoxal solution at 50 °C in a thermal cycler for 1 hour and precipitated with 3 M sodium acetate supplemented with glycogen (20 mg/ml) and cold ethanol at −80 °C overnight. After centrifuging and washing as described above, air-dried RNA pellets were reconstituted in 50 μl nuclease free water and mixed with 3 μl RNasin® Plus, 35 μl EndoV (0.5 μM, New England Biolabs, cat. No. M0305S), 25 μL 10× EndoVIPER binding and wash buffer, and 137 μL nuclease-free water, followed by 30 minutes of incubation at RT. The resultant reactions were mixed with anti-MBP magnetic bead (New England Biolabs, cat. No. E8037S) and incubated at RT for 2 hours with end-over end-rotation. After washing, beads were resuspended into 200 μL of 1× EndoVIPER elution buffer and incubated at 95 °C for 10 minutes to elute the RNAs. The resultant RNAs were purified with a Monarch RNA Cleanup Kit (New England Biolabs, cat. No. T2030S) and subjected to RT-qPCR analysis. The relative level of A-to-I RNA enrichment was defined as the ratio of the A-to-I RNA enrichments in VSMCs infected with Lenti-adarb1 or Lenti-Adarb1 (E396A) to VSMCs infected with Lenti-SCR, with Lenti-SCR set as 1.0.

**RNA electrophoretic mobility shift assay (RNA-EMSA)**

RNA-EMSAs were performed using LightShift Chemiluminescent RNA EMSA Kit (ThermoFisher, Catalog number: 20158) per manufacturers’ instructions. Briefly, nuclear proteins were extracted from serum-starved VSMCs using NE-PER™ Nuclear and Cytoplasmic Extraction Reagents (ThermoFisher, Catalog number: 78835) based on the protocol provided in the kit. VSMC nuclear proteins (25 μg) or mouse recombinant ADAR2 protein (500 ng, OriGene; CAT#: TP524167) was used for each binding reaction, which also contained Biotin-labelled RNA probe (1 nM), unlabelled RNA probe (100 or 200 nM), and/or anti-ADAR2 (1 μg) as indicated in the figure. All the binding reactions (20 μL) were incubated at room temperature for 25 minutes. After then, each binding reaction was mixed with 5μL of 5X Loading Buffer and loaded onto the pre-made and pre-run 6% polyacrylamide gel for electrophoresis (set to 100 V for 8 × 8 × 0.1cm gel). After electrophoretic transferred onto nylon membrane and crosslinked with a hand-held UV lamp equipped with 254nm bulbs, Biotin-labelled RNAs were detected by Chemiluminescence using a procedure similar to Western blot.

Below are the RNA probe sequences used in this study:

Biotinylated mmu-miR-22 RNA probe:

5´–AAGGUGACAGAACUGGGGUGGGAAGGUCU– 3´-Biotin

Unlabeled mmu-miR-22 RNA probe:

5´–AAGGUGACAGAACUGGGGUGGGAAGGUCU– 3´

Unlabeled mus snhg18 RNA probe:

5´–CAGAGUGCAAGCUCACCCAACUGGUGACU– 3´

**References:**

1.	Yang M, Chen Q, Mei L, Wen G, An W, Zhou X, Niu K, Liu C, Ren M, Sun K, Xiao Q and Zhang L. Neutrophil elastase promotes neointimal hyperplasia by targeting toll-like receptor 4 (TLR4)-NF-kappaB signalling. *British journal of pharmacology*. 2021;178:4048-4068.

2.	An W, Luong LA, Bowden NP, Yang M, Wu W, Zhou X, Liu C, Niu K, Luo J, Zhang C, Sun X, Poston R, Zhang L, Evans PC and Xiao Q. Cezanne is a critical regulator of pathological arterial remodelling by targeting beta-catenin signalling. *Cardiovascular research*. 2022;118:638-653.

3.	Chen Q, Yang F, Guo M, Wen G, Zhang C, Luong le A, Zhu J, Xiao Q and Zhang L. miRNA-34a reduces neointima formation through inhibiting smooth muscle cell proliferation and migration. *Journal of molecular and cellular cardiology*. 2015;89:75-86.

4.	Afzal TA, Luong LA, Chen D, Zhang C, Yang F, Chen Q, An W, Wilkes E, Yashiro K, Cutillas PR, Zhang L and Xiao Q. NCK Associated Protein 1 Modulated by miRNA-214 Determines Vascular Smooth Muscle Cell Migration, Proliferation, and Neointima Hyperplasia. *Journal of the American Heart Association*. 2016;5.

5.	Zhang L, Chen Q, An W, Yang F, Maguire EM, Chen D, Zhang C, Wen G, Yang M, Dai B, Luong LA, Zhu J, Xu Q and Xiao Q. Novel Pathological Role of hnRNPA1 (Heterogeneous Nuclear Ribonucleoprotein A1) in Vascular Smooth Muscle Cell Function and Neointima Hyperplasia. *Arteriosclerosis, thrombosis, and vascular biology*. 2017;37:2182-2194.

6.	Yang F, Chen Q, He S, Yang M, Maguire EM, An W, Afzal TA, Luong LA, Zhang L and Xiao Q. miR-22 Is a Novel Mediator of Vascular Smooth Muscle Cell Phenotypic Modulation and Neointima Formation. *Circulation*. 2018;137:1824-1841.

7.	Xiao Q, Zhang F, Grassia G, Hu Y, Zhang Z, Xing Q, Yin X, Maddaluno M, Drung B, Schmidt B, Maffia P, Ialenti A, Mayr M, Xu Q and Ye S. Matrix metalloproteinase-8 promotes vascular smooth muscle cell proliferation and neointima formation. *Arteriosclerosis, thrombosis, and vascular biology*. 2014;34:90-8.

8.	Zhang C, Chen D, Maguire EM, He S, Chen J, An W, Yang M, Afzal TA, Luong LA, Zhang L, Lei H, Wu Q and Xiao Q. Cbx3 inhibits vascular smooth muscle cell proliferation, migration, and neointima formation. *Cardiovascular research*. 2018;114:443-455.

9.	Luo Z, Wen G, Wang G, Pu X, Ye S, Xu Q, Wang W and Xiao Q. MicroRNA-200C and -150 play an important role in endothelial cell differentiation and vasculogenesis by targeting transcription repressor ZEB1. *Stem Cells*. 2013;31:1749-62.

10.	Yu X, Zhang L, Wen G, Zhao H, Luong LA, Chen Q, Huang Y, Zhu J, Ye S, Xu Q, Wang W and Xiao Q. Upregulated sirtuin 1 by miRNA-34a is required for smooth muscle cell differentiation from pluripotent stem cells. *Cell Death Differ*. 2014.

11.	Zhao H, Wen G, Huang Y, Yu X, Chen Q, Afzal TA, Luong LA, Zhu J, Shu Y, Zhang L and Xiao Q. MicroRNA-22 Regulates Smooth Muscle Cell Differentiation From Stem Cells by Targeting Methyl CpG-Binding Protein 2. *Arteriosclerosis, thrombosis, and vascular biology*. 2015.

12.	Liu SS, Maguire EM, Bai YS, Huang L, Liu Y, Xu L, Fauzi I, Zhang SQ, Xiao Q and Ma NF. A Novel Regulatory Axis, CHD1L-MicroRNA 486-Matrix Metalloproteinase 2, Controls Spermatogonial Stem Cell Properties. *Molecular and cellular biology*. 2019;39.

13.	Chen D, Zhang C, Chen J, Yang M, Afzal TA, An W, Maguire EM, He S, Luo J, Wang X, Zhao Y, Wu Q and Xiao Q. miRNA-200c-3p promotes endothelial to mesenchymal transition and neointimal hyperplasia in artery bypass grafts. *J Pathol*. 2021;253:209-224.

14.	He S, Yang F, Yang M, An W, Maguire EM, Chen Q, Xiao R, Wu W, Zhang L, Wang W and Xiao Q. miR-214-3p-Sufu-GLI1 is a novel regulatory axis controlling inflammatory smooth muscle cell differentiation from stem cells and neointimal hyperplasia. *Stem Cell Res Ther*. 2020;11:465.

15.	Zhang C, Niu K, Ren M, Zhou X, Yang Z, Yang M, Wang X, Luo J, Shao Y, Zhang C, Chen D, Gao S, Ge S, Wu Q and Xiao Q. Targeted Inhibition of Matrix Metalloproteinase-8 Prevents Aortic Dissection in a Murine Model. *Cells*. 2022;11.

16.	Xiao Q, Zhang F, Lin L, Fang C, Wen G, Tsai TN, Pu X, Sims D, Zhang Z, Yin X, Thomaszewski B, Schmidt B, Mayr M, Suzuki K, Xu Q and Ye S. Functional role of matrix metalloproteinase-8 in stem/progenitor cell migration and their recruitment into atherosclerotic lesions. *Circulation research*. 2013;112:35-47.

17.	Huang Y, Lin L, Yu X, Wen G, Pu X, Zhao H, Fang C, Zhu J, Ye S, Zhang L and Xiao Q. Functional involvements of heterogeneous nuclear ribonucleoprotein A1 in smooth muscle differentiation from stem cells in vitro and in vivo. *Stem Cells*. 2013;31:906-17.

18.	Fang C, Wen G, Zhang L, Lin L, Moore A, Wu S, Ye S and Xiao Q. An important role of matrix metalloproteinase-8 in angiogenesis in vitro and in vivo. *Cardiovascular research*. 2013;99:146-55.

19.	Xiao Q, Pepe AE, Wang G, Luo Z, Zhang L, Zeng L, Zhang Z, Hu Y, Ye S and Xu Q. Nrf3-Pla2g7 interaction plays an essential role in smooth muscle differentiation from stem cells. *Arterioscler Thromb Vasc Biol*. 2012;32:730-44.

20.	Wang G, Xiao Q, Luo Z, Ye S and Xu Q. Functional impact of heterogeneous nuclear ribonucleoprotein A2/B1 in smooth muscle differentiation from stem cells and embryonic arteriogenesis. *J Biol Chem*. 2012;287:2896-906.

21.	Zhao H, Wen G, Huang Y, Yu X, Chen Q, Afzal TA, Luong le A, Zhu J, Ye S, Zhang L and Xiao Q. MicroRNA-22 regulates smooth muscle cell differentiation from stem cells by targeting methyl CpG-binding protein 2. *Arteriosclerosis, thrombosis, and vascular biology*. 2015;35:918-29.

22.	Xiao Q, Zeng L, Zhang Z, Margariti A, Ali ZA, Channon KM, Xu Q and Hu Y. Sca-1+ progenitors derived from embryonic stem cells differentiate into endothelial cells capable of vascular repair after arterial injury. *Arterioscler Thromb Vasc Biol*. 2006;26:2244-51.

23.	Zeng L, Xiao Q, Margariti A, Zhang Z, Zampetaki A, Patel S, Capogrossi MC, Hu Y and Xu Q. HDAC3 is crucial in shear- and VEGF-induced stem cell differentiation toward endothelial cells. *J Cell Biol*. 2006;174:1059-69.

24.	Yu X, Zhang L, Wen G, Zhao H, Luong LA, Chen Q, Huang Y, Zhu J, Ye S, Xu Q, Wang W and Xiao Q. Upregulated sirtuin 1 by miRNA-34a is required for smooth muscle cell differentiation from pluripotent stem cells. *Cell death and differentiation*. 2015;22:1170-80.

25.	Torres M, Becquet D, Guillen S, Boyer B, Moreno M, Blanchard MP, Franc JL and Francois-Bellan AM. RNA Pull-down Procedure to Identify RNA Targets of a Long Non-coding RNA. *Journal of visualized experiments : JoVE*. 2018.

26.	Knutson SD, Arthur RA, Johnston HR and Heemstra JM. Selective Enrichment of A-to-I Edited Transcripts from Cellular RNA Using Endonuclease V. *Journal of the American Chemical Society*. 2020;142:5241-5251.

27.	Knutson SD and Heemstra JM. EndoVIPER-seq for Improved Detection of A-to-I Editing Sites in Cellular RNA. *Curr Protoc Chem Biol*. 2020;12:e82.

**Supplementary table S1**: Primer sets used in the present study

| **Gene names** | **Forward (5’-3’)** | **Reverse (5’-3’)** | **Application** |
| --- | --- | --- | --- |
| 18S/18s | AAACGGCTACCACATCCAAG | CCTCCAATGGATCCTCGTTA | Real-time RT-PCR |
| Gapdh | GGAGAGTGTTTCCTCGTCCC | ATGAAGGGGTCGTTGATGGC | Real-time RT-PCR |
| Snhg18 | TTTCGAACTCCCTTCCTCGC | TGGTTTGGGTTGAACTGCCT | Real-time RT-PCR |
| Acta2 | TCCTGACGCTGAAGTATCCGAT | GGCCACACGAAGCTCGTTATAG | Real-time RT-PCR |
| Tagln | GAT ATG GCA GCA GTG CAG AG | AGT TGG CTG TCT GTG AAG TC | Real-time RT-PCR |
| Cnn1 | GCGTCACCTCTATGATCCCAA | CCCAGACCTGGCTCAAAGAT | Real-time RT-PCR |
| Myh11 | AAG CAG CCA GCA TCA AGG AG | AGC TCT GCC ATG TCC TCC AC | Real-time RT-PCR |
| Sp1 | ACAGGGTGCCAATGGCTGGC | GCCCACCAGAGACTGTGCGG | Real-time RT-PCR |
| Mecp2 | GGCTGTGGTAAAACCCGTCCG | GGCTTGTCTCTGAGGCCCTGGA | Real-time RT-PCR |
| Evi1 | GCAGACATTGCGCCTGGGGAA | CTCACAGCGGTGCTGCCGTT | Real-time RT-PCR |
| Hdac4 | TGCGTCCTGCCTTGGTGCTC | GCGCGCTGTCCTGTGTTCCA | Real-time RT-PCR |
| Pcna | TTGCACGTATATGCCGAGACCT | ATTGCCAAGCTCTCCACTTGC | Real-time RT-PCR |
| U6/u6 snoRNA | GATGACACGCAAATTCGTG | miRNA universal reverse primer (Invitrogen, A11193-051) | Real-time RT-PCR |
| miR-22-3p | AAGCTGCCAGTTGAAGAACTGT | miRNA universal reverse primer (Invitrogen, A11193-051) | Real-time RT-PCR |
| miR-34a-5p | GGCAGTGTCTTAGCTGGTTGT | miRNA universal reverse primer (Invitrogen, A11193-051) | Real-time RT-PCR |
| miR-214-3p | CAGGCACAGACAGGCAGT | miRNA universal reverse primer (Invitrogen, A11193-051) | Real-time RT-PCR |
| miR-142a-5p | CATAAAGTAGAAAGCACTACT | miRNA universal reverse primer (Invitrogen, A11193-051) | Real-time RT-PCR |
| Cbx3 | GAACGAATAATCGGCGCCA | ATGTTCGCCTCCTTTGCCA | Real-time RT-PCR |
| Hnrnpa1 | TTCATCCAGTCAGAGAGGTCGC | TGAAGTTCCCTCCTCGACCAA | Real-time RT-PCR |
| Mmp8 | ATCAGCAGAGGTATGGGCAC | TCTGCCTGGGAACTTATTGG | Real-time RT-PCR |
| Cezanne | TGTCCGATTGGCCAGTATAA | ACAGTGGGATCCACTTCACA | Real-time RT-PCR |
| Elane | CAGAGGCGTGGAGGTCATTT | CTACCTGCACTGACCGGAAA | Real-time RT-PCR |
| Pri-miR-22 | AAAGGGGCACAAAGCAAGTG | CAGGAAAGCTGGGTGACAGG | Real-time RT-PCR |
| Pre-miR-22 | ACCTGGCTGAGCCGCAGTAG | AGGGGCAGAGGGCAACAGTTC | Real-time RT-PCR |
| Pre-miR-124 | ACCCATAAAGTAGAAAGCAC | CATCCATAAAGTAGGAAACAC | Real-time RT-PCR |
| Adarb1 | CGAGGGTATTCCGCTCTCCA | CCCTGGTGTTTTCCTTCGCT | Real-time RT-PCR |
| SNHG18 | GCCCCTCCACTTGGATTTCA | TGGGCTGCCACACTAAAGAG | Real-time RT-PCR |
| ACTA2 | TGAGCGTGGCTATTCCTTCGT | GCAGTGGCCATCTCATTTTCAA | Real-time RT-PCR |
| TAGLN | GGCTGAAGAATGGCGTGATT | TCTGCTTGAAGACCATGGAGG | Real-time RT-PCR |
| MYH11 | GCGTCCATGCCAGATAACACA | TACCACATCTCGCCCAACCTT | Real-time RT-PCR |
| CNN1 | ATTTTTGAGGCCAACGACCTG | CCCACGTTCACCTTGTTTCCT | Real-time RT-PCR |
| Pdgfb | GCTCCGTCTACGCGTCC | GAATGGGATCCCCCTCGG | Real-time RT-PCR |
| Tgfb1 | ACTGGAGTTGTACGGCAGTG | GGGGCTGATCCCGTTGATT | Real-time RT-PCR |
| Snhg18 promoter | GAGTGGGCACAGCGTGAG | TCCTGTGCTCCCCGCC | CHIP assay |
| Snhg18 intron | TCCTGGTATTGGCAGGTTGC | AGCTATTACCCGGTCCCTGT | CHIP assay |
| Snhg18 | TACCACGCCTTTACACTGGC | ATAAAACCGAAGCAGCACCG | Real-time RT-PCR (RNA pull-down) |
| Snhg18 | TTTCGAACTCCCTTCCTCGC | TGGTTTGGGTTGAACTGCCT | Real-time RT-PCR (RIP) |

**Supplementary table S2**: Potential interactions of snhg18 with RNA binding proteins predicted by ATtRACT (<https://attract.cnic.es/seqsearch>)

**Supplementary Figures:**

**Figure S1. Single cell RNA sequencing and transcriptomic analysis of SNHG18 expression in murine and human arteries**

**(A)** Focused analysis of scRNA-Seq dataset of guide-wire injured mouse femoral arteries (GSE182232) showed that snhg18 gene expression was highly and positively associated with VSMC contractile genes (Myh11, Cnn1, Tagln, Acta2) in injured arteries. (B) scRNA-Seq analysis. scRNA-seq datasets (GSE155513) of aortic cells isolated from aortas of LDLR^-/-^ mice fed with high-fat diet for various time points (0, 8, 16, 26 weeks) were downloaded and processed for reanalysis. Violin plots showing the decreased expression levels of VSMC-specific genes (Myh11, Cnn1, Tagln, Acta2) and snhg18 in all aortic cells and VSMCs in mouse atherosclerotic arteries. (C) Re-analysis of microarrays dataset (GSE100927) showing the decreased expression levels of SNHG18 in human atherosclerotic lesions of carotid (AS_CA), femoral (AS_FA), and infra-popliteal (AS_IPA) arteries. *P<0.05 (versus control). (D) RNA-seq analysis of stable and unstable section of human atherosclerotic plaques (GSE120521) showing a trend of decreased expression levels of SNHG18 in unstable human atheroscleorisis. NS, non-significant.

**Figure S2.** **Fluorescence in situ hybridisation (FISH) detection of snhg18 in femoral artery**

Sham (uninjured FA) or injured (injured FA, day 7 post-injury) femoral arteries were collected and subjected to FISH analysis using a scrambled control (SCR) or snhg18-specific probe, respectively. Immunofluorescence (IF) staining was simultaneously conducted with SMA antibody or IgG control. The data presented here are representatives images of three (n=3) independent experiments.

**Figure S3. Sp1 is downregulated in injured femoral arteries.**

Sham (uninjured) and injured femoral arteries (FA) collected at day 3, 7, 14, and 28 post-injury were subjected to RT-qPCR (A) and IF staining (B-C), respectively. Data in A represent the mean±SEM (n=5), where 3 injured femoral arteries were pooled as one experiment. **P*<0.05 (versus uninjured FA, one-way ANOVA with a post hoc test of Tukey’s analysis). The data presented in (B-C) are representatives images (B) or mean±SEM (C) of five (n=5) mice. **P*<0.05 (versus uninjured FA, unpaired *t*-test).

**Figure S4. Functional involvement of SMAD3/4 in TGFβ1-mediated snhg18 regulation in VSMCs**

(A) Schematic illustration of DNA sequence for mouse snhg18 gene promoter and potential binding sites for transcription factor SMAD3/4. +1 indicates translation initiation site. (B) Luciferase activity analysis of mouse snhg18 gene promoters. Left panel, schematic diagram of wild-type (pGL4-snhg18-WT) and SMAD binding site deletions (pGL4-snhg18-d1, -d2, -d1/2) snhg18 gene promoter reporters. Right panel, luciferase activity assays with the indicated reporters. Data and error bars represent the mean±SEM (n=5). *P<0.05 (versus vehicle); ^#^P<0.05 (versus pGL4-snhg18-WT) (two-way ANOVA with a post hoc test of Tukey’s analysis).

**Figure S5. Cellular location of Snhg18 in VSMCs.**

**(A)** Snhg18 is mainly enriched in nucleus in VSMCs. Nuclear and Cytoplasmic RNA was isolated from serum-starved VSMCs using the SurePrep™ Nuclear or Cytoplasmic RNA Purification Kit Product (Cat. # BP2805-25), and subjected to RT-qPCR analysis. (B) Fluorescence in situ hybridisation (FISH). Serum-starved VSMCs were subjected to FISH analysis using a scrambled control or snhg18-specific probe, respectively. The data presented here are representatives (B) or mean±SEM (A) of five (n=5) independent experiments. *P<0.05 (vs. cytosol) (unpaired *t*-test).

**Figure S6. Snhg18 controls PDGF-BB induced VSMC migration.**

VSMCs were treated as described in Figure 3, and subjected to transwell migration assay. (A-B) Snhg18 overexpression inhibited VSMC migration. (C-D) Snhg18 inhibition increased VSMC migration. Note: In transwell migration, 30 ng/ml PDGF-BB was used as chemoattractant for starved VSMCs, since only a few cells migrated through the insert with vehicle control, 10 ng/ml PDGF-BB or TGFβ1 (5 ng/ml). The data presented here are representatives (A and C) or mean±SEM (B and D) of five (n=5) independent experiments. *P<0.05 (vs. Lenti-SCR or LNA-SCR) (unpaired *t*-test).

**Figure S7. The regulatory effect of snhg18 overexpression on VSMC migration and contraction was rescued by miR-22-3p inhibition.**

VSMCs were treated as described in Figure 4C-F, and subjected to transwell migration assay in response to 30 ng/ml PDGF-BB (A-B) or collagen-based VSMC contraction assay (C-D), respectively. Representative images (C) and percentage (%, D) of VSMC contraction were presented here. The data presented here are representatives (A & C) or mean±SEM (B & D) of five (n=5) independent experiments. *P<0.05 (vs. Lenti-SCR), ^#^P<0.05 (miR-22 inhibitor vs. miR Ctrl) (two-way ANOVA with a post hoc test of Tukey’s analysis).

**Figure S8. The regulatory effect of snhg18 overexpression on VSMC gene expression and proliferation was rescued by miR-22-3p inhibition in the presence of TGFβ1.**

VSMCs infected with Lenti-SCR or Lenti-snhg18 were transfected with a control (miR Ctrl) or miR-22-3p inhibitor, respectively. After then, cells were subjected to serum starvation for 24 hours, followed by TGFβ1 treatment (5 ng/ml) for additional 24 hours. Cells were collected and subjected to RT-qPCR (A) and CCK-8 (B) analysis, respectively. The data presented here are mean±SEM of five (n=5) independent experiments. *P<0.05 (vs. Lenti-SCR), ^#^P<0.05 (vs. miR Ctrl) (two-way ANOVA with a post hoc test of Tukey’s analysis).

**Figure S9. Potential interaction between snhg18 and mmu-miR-22 predicted RNA hybridization.**

**Figure S10. Detection of A-to-I editing in pre-miR-22.**

**(A)** Low level of A-to-I RNA editing in miR-22 precursor (Pre-miR-22). VSMCs were infected with control (Lenti-SCR), wild type (Lent-adarb1), or a catalytically inactive adarb1 variant (Lenti-adarb1 (E396A)) overexpression lentivirus and subjected to serum starvation for 24 hours, followed by Endonuclease V immunoprecipitation enrichment (EndoVIPER) in the presence of 5 mM Ca2^+^ to enrich A-to-I edited transcripts. Purified and enriched RNA was subjected to RT-qPCR analysis to detect miR-142 and -22 precursor, respectively. (**B)** RT-qPCR detection of Adarb1, miR-142, pre-miR-22 and miR-22-3p. VSMCs infected with the indicated lentivirus were subjected to serum starvation for 24 hours. Total RNA was extracted and subjected to RT-qPCR analysis. Data and error bars represent mean±SEM of five (n=5) independent experiments. *P<0.05 (vs. Lenti-SCR), ^#^P<0.05 (vs. Lenti-adarb1) (one-way ANOVA with a post hoc test of Tukey’s analysis).

**Figure S11. TGFβ1 promotes miR-22 biogenesis in VSMCs.**

Serum-starved VSMCs were treated with 5 ng/ml TGFβ1 for 24 hours, followed by RT-qPCR (A-B) and RNA immunoprecipitation (RIP, C-D) assays, respectively. (A) TGFβ1 increased expression level of precursor (pre-miR-22) but not the primary (Pri-miR-22) miR-22 in VSMCs. (B) Adarb1 gene expression was not impaired by TGFβ1. (C-D) RIP assay with IgG control or ADAR2 antibody showing TGFβ1 increased ADAR2 binding to snhg18 transcript (C), but decreased ADAR2 binding to pri-miR-22 transcript. Data and error bars represent mean±SEM of five (n=5) independent experiments. *P<0.05 (vs. vehicle), ^#^P<0.05 (vs. IgG) (two-way ANOVA with a post hoc test of Tukey’s analysis).

**Figure S12. Per-vascular delivery of lentiviral particles and miRNAs.**

After injury, 100µl of 30% pluronic gel contained 2.0 x 10^6^ lentiviral particles (Lenti-GFP or Lenti-snhg18) and/or 2.5 nmol miRNA inhibitor (LNA-SCR or LNA-miR-22), was immediately applied and packed around the injured vessel. Injured segments of femoral arteries and other tissues (heart, lung, liver) collected at day 7 post-injury were prepared and subjected to IF staining with GFP antibody (A) or RT-qPCR (B & C) analysis, respectively. Data presented in (A & C) are representative images (A) or mean±S.E.M (C) from six mice (n=6), while data in (B) are mean±S.E.M from three independent experiments (note: injured femoral arteries were collected and carefully separated into two portions: media/neointima (Med&NI) and adventitia. RNAs from each portion of 6 arteries were pooled as 1 experiment in (B).
